# Nitrate nitrogen uptake and metabolism in *Mikania micrantha* stem: insights into enhanced growth and invasiveness

**DOI:** 10.3389/fpls.2025.1525303

**Published:** 2025-05-02

**Authors:** Minling Cai, Lihua Chen, Minghao Chen, Weiqian Ke, Dongguang Wang, Changlian Peng

**Affiliations:** ^1^ College of Life Science, Huizhou University, Huizhou, China; ^2^ Guangzhou Key Laboratory of Subtropical Biodiversity and Biomonitoring, Guangdong Provincial Key Laboratory of Biotechnology for Plant Development, College of Life Sciences, South China Normal University, Guangzhou, China; ^3^ Key Laboratory of Vegetation Restoration and Management of Degraded Ecosystems, South China Botanical Garden, Chinese Academy of Sciences, Guangzhou, China

**Keywords:** invasive plant, NO_3_⁻-N, RNA-seq, gene, stem

## Abstract

The increasing atmospheric nitrogen deposition, characterized by a rising proportion of nitrate nitrogen (NO_3_⁻-N), is exacerbating the spread of invasive plant species. Despite this trend, the response mechanisms of *Mikania micrantha*, a highly invasive plant, to NO_3_⁻-N remain poorly understood. This study investigates the unique adaptation strategies of *M. micrantha* to elevated NO_3_⁻-N levels, providing novel insights into its invasive success under changing nitrogen deposition patterns. Field experiments showed that *M. micrantha* rhizosphere soil contained higher NO_3_^–^N content and protease activity compared to companion plants (*Paederia scandens*, *Ipomoea nil*, and *Ipomoea cairica*). Both roots and stems of *M. micrantha* had higher NO_3_^–^N content and demonstrated stronger nitrogen metabolism capabilities. Pot experiments further showed that increasing NO_3_⁻-N concentrations (0 mM–40 mM) significantly promoted *M. micrantha* growth, with optimal phenotypic responses (main stem length, leaf number, branch number, and biomass) observed at 5 mM NO_3_⁻-N. Nitrogen metabolism enzyme assays revealed that nitrate reductase (NR), nitrite reductase (NiR), glutamate dehydrogenase (GDH), and free amino acid content increased progressively with NO_3_⁻-N concentration. Transcriptome sequencing and qPCR analyses identified upregulation of key genes related to transcription factors, nitrate transporter-related, nitrogen metabolism enzyme, and amino acid synthesis pathway. These findings demonstrate that *M. micrantha* employs a multifaceted strategy to exploit elevated NO_3_⁻-N conditions: enhanced NO_3_⁻-N uptake from soil, efficient transport to stems, and robust nitrogen metabolism facilitated by coordinated gene expression. This study reveals the adaptation mechanisms of *M. micrantha* to NO_3_⁻-N enrichment, offering critical insights for predicting and managing invasive species responses to global atmospheric nitrogen deposition changes. The results highlight the importance of considering nitrogen composition, rather than just quantity, in invasive species management strategies.

## Introduction

Since the industrial revolution, human activities such as fertilizer application and fossil fuel combustion have significantly increased atmospheric nitrogen deposition (Stevens et al., 2011). China has become one of the three major high nitrogen deposition areas in the world. Data indicates that anthropogenic nitrogen production was 18.3 Tg N in 1980, which doubled by 2010 to 53.9 Tg N (Gu et al., 2015). Nitrogen is an essential nutrient for plant growth and development, with its demand significantly increasing compared to other elements (Toor et al., 2021). While high nitrogen deposition typically increases soil nitrogen availability and stimulates plant growth (Dong et al., 2022), excessive nitrogen can cause a range of ecological problems. These include impacts on human health, alterations in biogeochemical cycles, shifts in ecosystem structure and function, and even the potential extinction of species (Chen et al., 2020; Jia et al., 2020; Zhao et al., 2021). Ammonium-nitrogen (NH_4_^+^-N) and nitrate-nitrogen (NO_3_^–^N) are the two main inorganic nitrogen forms absorbed by plants (Wang et al., 2021). NO_3_^–^N deposition primarily originates from industrial and transportation fossil fuel emissions (Galloway et al., 2004), whereas NH_4_^+^-N deposition is largely derived from artificial fertilizers and livestock farming (Behera et al., 2013). Research indicates that the RNHx/NOy ratio in nitrogen deposition has been decreasing in China, due to an ongoing increase in NO_3_^–^N deposition alongside a decrease in NH_4_^+^-N deposition. This marks a transition from the previous NH_4_^+^-dominant nitrogen deposition mode to a new mode where NH_4_^+^-N and NO_3_^–^N deposition contribute equally (Zhu et al., 2015; Yu et al., 2019). Therefore, in the context of global nitrogen deposition, especially the alteration of nitrogen deposition components (RNHx/NOy), controlling the invasion of exotic plants has become an urgent priority (Shuvar and Korpita, 2021).

Biological invasions not only threaten the abundance and diversity of native species but also alters the carbon and nitrogen cycles in ecosystems, thereby affecting their structure and function. Increased nitrogen deposition significantly accelerates the growth and spread of nitrophilous plants, which can lead to the exclusion of plants with lower nitrogen requirements from the community, ultimately causing their decline or even extinction. Numerous studies indicated that nitrogen fertilization favored the growth and invasion of exotic species (Bobbink et al., 2010; Antonio and Mack, 2006). Eller and Oliveira (2017) found that the invasive plant *Melinis minutiflora* exhibited a stronger competitive advantages and interfered with the uptake of nitrogen by native specie *Aristida adscensionis*, making it more beneficial in high-nitrogen environments. Additionally, Peng et al. (2019) reported that nitrogen addition increased the leaf lifespan, plant height, and early flowering of the invasive plant *Solidago canadensis*. Invasive species typically exhibit excellent phenotypic plasticity and resource use efficiency compared to native species (Vaz-Pinto et al., 2014), enabling them to survive even under stressful conditions. Furthermore, studies suggested that different plants exhibit varied responses and preferences for different nitrogen forms (Yousaf et al., 2016; Qian et al., 2021; He et al., 2021). The invasive species *Wedelia trilobata* exhibited better adaptation to environmental conditions with an NH_4_^+^-N/NO_3_^–^N ratio of 2:1 through faster growth and antioxidant defense system compared to *Wedelia chinensis* (Huang et al., 2022). Under elevated NH_4_^+^-N levels, the invasive species *Phyllostachys edulis* demonstrated superior growth, nitrogen uptake and NH_4_^+^-N tolerance compared to *Castanopsis fargesii*, thereby facilitating its expansion (Zou et al., 2020). The invasive plant *Flaveria bidentis* exhibited an increase in plant height and branching under high ammonium cultivation conditions (Huangfu et al., 2016). Conversely, some studies suggested that invasive plants such as *Amaranthus retroflexus* and *Bidens pilosa* exhibit better growth advantages in habitats with higher NO_3_^–^N level (Wang et al., 2018; Chen and Chen, 2019). However, most researchers have primarily focused on the physiological and ecological responses of invasive plants to nitrogen deposition and its various forms, leaving the invasion mechanisms of exotic plants remain unclear.

*Mikania micrantha* (Asteraceae family), native to Central and South America, has become widespread in Asia and the Pacific Islands and is listed as one of the world’s top 100 most threatening alien invasive species. Due to its rapid growth and strong adaptability, it can quickly colonize invaded areas, causing significant damage to local ecosystems and severe economic losses (Day et al., 2016). The rapid growth of stem is an important characteristic of *M. micrantha*, with certain photosynthetic activity (Cai et al., 2023; Liu et al., 2020) and stress resistance (Chen et al., 2024; Zhang et al., 2019), playing an important role in its rapid invasion process. Fang et al. (2021) found that *M. micrantha* has expanded rapidly in terms of invaded area over the past 30 years. It is predicted that in the 2050s and 2070s, *M. micrantha* will continue to rapidly spread from Yunnan and Guangdong provinces towards the northern regions and inland areas. Therefore, understanding the mechanisms facilitating the rapid growth of *M. micrantha* is crucial for the effective control of invasive plant species. Studies have shown that as CO_2_ concentrations and nitrogen deposition rose, the invasive potential of *M. micrantha* increased (Zhang et al., 2016). Compared to native plants *Polygonum chinense* and *Paederia scandens*, *M. micrantha* demonstrated strong competitive resource utilization capabilities in terms of nitrogen acquisition and soil nitrogen mineralization (Yu et al., 2021). Liu et al. (2020) found that NH_4_^+^-N significantly increased in soil after the invasion of *M. micrantha*, but NO_3_^−^-N content significantly decreased. These results suggested that *M. micrantha* possesses a strong ability to acquire nitrogen, potentially exhibiting preferential selection for the NO_3_^–^N. Hence, we propose a hypothesis that, in the context of increasing global nitrogen deposition, particularly with the continuous increase of NO_3_^−^-N deposition, the rapid growth of *M. micrantha* may enhance nitrogen utilization efficiency in the main form of NO_3_^−^ by regulating the expression levels of key genes or proteins involved in NO_3_^−^-N absorption, thereby accelerating its diffusion trend to the north and inland. Our study aims to improve the management of *M. micrantha* invasion control in the context of global change in the future.

## Materials and methods

### Plant collection and cultivation

The naturally growing *M. micrantha* and associated plants (*P. scandens*, *Ipomoea nil*, and *Ipomoea cairica*) were used for the identification of nitrogen absorption patterns. Samples were collected from the South China Normal University botanical garden in Guangzhou, China (23°10′N, 113°21′E) during growing season (July–August). Stems from the first to the fourth internodes of *M. micrantha* and its associated plants were collected for physiological data determination. The rhizosphere soil of plant was collected and stored at 4°C.

Control experiment starting from seed germination was carried out to observe the response of stems under different NO_3_^−^-N concentrations. Seeds of *M. micrantha* and *P. scandens* were placed in a constant temperature incubator (12h/12h light-dark cycle, light intensity of 100–120 mmol m^-2^ s^-1^, day/night temperature of 25 ± 1 °C). After 1–2 weeks of cultivation, healthy seedlings were selected and transplanted into pots. The cultivation substrate consisted of a mixture of *Arabidopsis* soil and vermiculite in a 3:1 ratio. After one month of cultivation, *M. micrantha* and *P. scandens* with consistent growth were selected for nutrient solution cultivation experiments. Samples with similar growth were divided into 6 groups, and were treated with modified Hoagland nutrient solutions with 0, 0.5, 5, 10, 20, and 40 mM NO_3_^−^-N respectively (**Table 1**). Fifteen repetitions were set for each group. Each group was treated for 30 days, with treatment every 3 days.

**Table 1:** Modified hoagland nutrient solution.

Nutrition	Reagent	Concentation (g L^-1^)	Dosage (mL)					
			0 mM	0.5 mM	5 mM	10 mM	20 mM	40 mM
Macroelement	KNO_3_	50.55	0	1	10	20	40	80
	KCl	37.28	80	79	70	60	40	0
Microelement	CaCl_2_	22.2	10					
	KH_2_PO_4_	5.444	10					
	MgSO_4_·7H_2_O	24.65	10					
	H_3_BO_3_	0.185	1					
	MnSO_4_·H_2_O	0.0845	1					
	CuSO_4_·5H_2_O	0.0250	1					
	ZnSO_4_·7H_2_O	0.0288	1					
	(NH_4_)_6_Mo_7_O_24_·4H_2_O	0.124	1					
	FeSO_4_·7H_2_O	13.9	1					
	EDTANa_2_·2H_2_O	18.6	1					

### Analysis of basic growth indicators

Plant main stem length, leaf number, branching number, and axillary bud number were measured using a ruler every 3 days. Phenotypic changes were documented using a camera. Subsequently, the roots, stems, and leaves of each plant were separated, dried at 75°C for 72 hours, and weighed to record the biomass.

### Soil physicochemical properties

Soil physicochemical properties, including pH, moisture content, NH_4_^+^–N and NO_3_^−^–N levels, and protease enzyme activity, were measured as follows: (1) Measurement of pH value and moisture content: Following the method by Yu et al. (2021), 5 g of fresh rhizosphere soil was mixed with 0.01 M CaCl_2_ solution, shaken, and extracted for 30 minutes. Soil pH was measured directly measured using a pH meter (ST3100, Ohaus Instruments (China) Co., Ltd.), and moisture content was determined after drying at 60°C. (2) Nitrogen content determination: Inorganic nitrogen forms (NH_4_^+^ –N and NO_3_^−^–N) were extracted from fresh soil using 2 M KCl solution (Yu et al., 2021). NH_4_^+^–N was quantified using the indophenol blue colorimetric method, with absorbance measured at 625 nm. NO_3_^−^–N was measured directly via UV spectrophotometry. Standard curves were prepared using ammonium sulfate ((NH_4_)_2_SO_4_) for NH_4_^+^–N and potassium nitrate (KNO_3_) for NO_3_^−^–N. (3) Protease enzyme activity determination: Protease enzyme activity was determined using the sodium caseinate method (Ladd and Butler, 1972). Fresh soil (2.5 g) was incubated with Tris buffer (pH=8.1) and 2% sodium caseinate solution at 50°C for 2 hours. After adding 15% TCA solution and filtering, the supernatant was mixed with alkaline reagent and Folin’s reagent. Absorbance was measured at 700 nm, and a standard curve was prepared using sodium caseinate.

### Determination of nitrogen content and nitrogen metabolism enzyme activities in plant tissues

The nitrogen content (NO_3_^−^–N and NH_4_^+^–N) and key enzymes activities related to nitrogen metabolism, including nitrate reductase (NR), nitrite reductase (NiR), glutamine synthetase (GS), glutamate synthase (GOGAT) in plant tissues, were measured using reagent kits from Suzhou Koming Biotechnology Co., Ltd. The activity of GDH was determined according to the method described by Zhou et al. (2015). Fresh stem segments (third or fourth) and lateral roots (0.1 g) were homogenized in 1 mL extraction buffer (Suzhou Koming Biotechnology Co., Ltd.) with quartz sand in an ice bath. The homogenate was centrifuged at 10,000 ×g and 4°C for 10 min, and the supernatant was stored at 4°C for analysis. The 3 mL reaction mixture consisted of 2.6 mL of stock solution (115.4 mmol L^-1^ pH=8 Tris-HCl buffer, 23.1 mmol L^-1^ a-Ketoglutaric acid, 231 mmol L^-1^ NH_4_Cl), 0.1 mL CaCl_2_, and 0.1 mL ddH_2_O. After incubating at 30°C for 10 minutes, 0.1 mL of NADH and 0.1 mL of the supernatant were added. Absorbance values at a wavelength of 340 nm were measured every minute using a UV-2450 spectrophotometer (Shimadzu, Tokyo, Japan). GDH activity were expressed as nmol min^-1^ g^-1^ FW.

### Determination of free amino acid content

The free amino acid content was determined following Doi et al. (1981) with slight modifications.

Fresh stem segments (third or fourth) and lateral roots (0.1 g) were homogenized in 1.5 mL of 10% acetic acid with quartz sand in an ice bath. The homogenate was centrifuged at 12,000 × g and 4°C for 10 min to obtain the supernatant. A volume of 1 mL of the supernatant was mixed with 1 mL sodium acetate buffer (pH=5.4) and ninhydrin reagent, then heated in a boiling water bath at 100°C for 15 min. After cooling, 3 mL of 60% ethanol was added, followed by thorough mixing. The absorbance at OD_570nm_ was measured using a spectrophotometer.

### Analysis of transcriptome sequencing

To analyze the gene expression patterns of *M. micrantha* stems in response to different NO_3_^−^-N concentrations, the third to fourth internodes of stems at 0 mM and 5 mM were selected for transcriptomic sequencing. Total RNA extraction and library construction were conducted using the Spin Column Plant Total RNA Purification Kit (Sangon, Shanghai, China.) and the HiPure Total RNA Mini Kit (Magen), respectively. RNA integrity and purity were assessed using agarose gel electrophoresis and UV spectrophotometry. Qualified RNA were subjected to library construction using the Novogene NGS RNA Library Prep Kit. The quality of the constructed libraries was evaluated using the Agilent 2100 Bioanalyzer and ABI StepOnePlus Real-Time PCR System, followed by sequencing on the IIIumina HiSeq 4000 platform after passing quality control. Clean reads were aligned to the reference genome using HISAT2 software. Differential gene expression analysis was performed using DESeq2 software, with the criteria for selecting differential genes set as an absolute fold change ≥2 and Padj value ≤0.05. DEGs were functionally annotated using databases such as Nonredundant protein (Nr), Nonredundant nucleotide (Nt), Swiss-Prot, Kyoto Encyclopedia of Genes and Genomes (KEGG), and Gene Ontology (GO).

### Quantitative real time RT-PCR of key gene expression

Following the protocol of Cai et al. (2022), fresh stem segments (0.1 g) were ground in liquid nitrogen. Total RNA was isolated using a Quick RNA isolation Kit (Huayueyang) according to the manufacturer’s protocol and was quantified using a spectrometer (NanoDrop). cDNA was synthesized using the TopScript-RT-DryMIX (dT18) kit (Takara, Tokyo, Japan). The relative expression levels of key genes were analyzed was estimated by qRT-PCR with a SYBR Green master mix (SYBR Green Premix Ex Taq, Takara, Japan) in a Bio-Rad CFX96 Real-Time PCR Detection System (Bio-Rad Laboratories Inc., Hercules, CA, USA). The PCR program was run as follows: 95°C for 30 seconds, followed by 39 cycles of 95°C for 5 s, 60°C for 34 s, and 65°C for 5 s, with a final extension step at 95°C for 50 seconds. The reference gene used was *18S*, and the relative expression of the candidate genes were calculated using the 2^–ΔΔCT^ method. The specific primers for reference gene and candidate genes were listed in **Table 2**.

**Table 2:** Gene-specific primers used for qRT-PCR.

Gene name	Forward primer (5’-3’)	Reverse primer (5’-3’)
*18s*	GTCGGGGGCATTCGTATTTC	CGGCATCGTTTATGGTTGAG
*NPF5*	TCAGCCGTCTTGACCACTTC	ACCTGAGCTTTCCTCTGCAC
*NPF6*	GGGACAGGCACACTAGCATT	ATTTAAGACCGCCAGTCCCG
*CLC-g*	TGCTATCGTCGGCTCCAATC	CTGTTGCGCCAATCTTGCTT
*SLAH3*	ATATGTCTCGGCGTCAGCAG	GGACGGAGATGCACCATAGG
*HY5.1*	CCGAAGGTTCCCGGAGAAAA	TTGACTCGCACCTCCAACTC
*LBD38*	GATACAGTCCTTCGAGGCGG	ACGGACTTCCCTGACGTAGA
*NR1.1*	ACGACTGGTCCGTGGAGATA	GTTGCCGGGAACTCTCTTGA
*NiR1.2*	AGTTCCACCAGGCTGTATGC	ATCGGTTCACCGCCAATCTT
*GS*	ATTTCCGCTGGTGACGAGTT	TCCAGCACCATTCCAGTCAC
*GDH*	ACGACTTGCTGGTTTCCGAT	GCAGCAGCATGCTTGTATCC

### Data analysis

The statistical analyses, including significant difference analysis and regression analysis, were conducted using IBM SPSS Statistics 19.0 (IBM, NY, USA). The results were presented as mean ± standard error. Independent sample t-tests were utilized for the significant difference analysis between each concentration group (0.5–40 mM) and the control group (0 mM), with * indicating a significant difference (0.01<*p*<0.05), ** indicating an extremely significant difference (*p*<0.01), and ns indicating no significant difference (*p*>0.05). Data visualization was performed using SigmaPlot 14.0 (Systat Software, San Jose, CA, USA). Additionally, a correlational analysis (Pearson correlation) was applied to measure the correlations among between *M. micrantha* and *P. scandens* under different concentrations of NO_3_^−^–N (0–40 mM). Principal component analysis (PCA) was carried out to describe the degree of association and determine possible factors that affect the biomass in both plant under NO_3_^−^–N concentration. PCA analysis was performed using Origin 2018 software (OriginLab, Northampton, MA, USA).

## Results

### Differences in nitrogen absorption, utilization, and metabolism of *M. micrantha* and its companion plants

The analysis of rhizosphere soil properties (**Table 3**) showed that *I. cairica* exhibited significantly higher soil moisture content compared to *M. micrantha*, *I. nil*, and *P. scandens* (*p*<0.05). The NO_3_^−^–N content in *M. micrantha* was significantly higher compared to *P. scandens*, *I. nil*, and *I. cairica* by 2.3-, 2.5-, and 2-fold, respectively, as evidenced by the non-overlapping 95% confidence intervals (CI). Conversely, NH_4_^+^–N content in *I. cairica* and *I. nil* were notably higher than that in *M. micrantha* and *P. scandens*. The protease activity was significantly higher in *M. micrantha*, surpassing *I. cairica* and *I. nil* by approximately 1.3-fold but only slightly exceeding *P. scandens*.

**Table 3:** Comparison of nitrogen absorption characteristics in soil, roots, and stems of *M. micrantha* and its associated plants under field conditions (mean ± standard error, n=5, numbers in parentheses are 95% CI).

	Indices	*M. micrantha*	*I. cairica*	*I. nil*	*P. scandens*
Soil	Moisture content(%)	21.17±0.19b (20.68, 21.66)	26.12±1.59a (22.04, 30.21)	21.73±0.51b (20.53, 22.93)	22.85±0.99b (20.31, 25.39)
	NH_4_^+^–N (μg g^-1^ Fw)	0.22±0.01bc (0.20, 0.25)	0.34±0.02ab (0.23, 0.45)	0.41±0.07a (0.18,0.64)	0.16±0.08c (0.13, 0.19)
	NO_3_^-^–N (μg g^-1^ Fw)	0.65±0.03a ( 0.57, 0.72 )	0.28±0.05b ( 0.13, 0.43)	0.26±0.18b ( 0.20, 0.31)	0.31±0.06b ( 0.13, 0.49)
	Protease (μg g^-1^ h Fw)	26.88±0.40a ( 25.16, 28.59)	20.36±2.02b (15.18, 25.55)	20.58±1.10b (17.53, 23.63)	25.37±0.93ab (21.36, 29.38)
Root	NH_4_^+^–N (μg g^-1^ Fw)	22.37±0.95b (19.33, 25.41)	26.31±0.73ab (24.44, 28.17)	25.23±0.91b (22.33, 28.13)	32.05±3.73a (21.68, 42.42)
	NO_3_^-^–N (μg g^-1^ Fw)	123.14±8.35a (99.95, 146.32)	118.71±9.04a (89.95, 147.47)	53.97±3.06b (46.12, 61.83)	62.41±3.99b (45.24, 79.59)
	NR (nmol min^-1^ g^-1^ Fw)	225.05±11.74a (187.69, 262.41)	233.09±15.39a (184.11, 282.07)	106.90±3.56c (95.56, 118.24)	172.32±6.56b (154.12, 190.53)
	GS (μmol min^-1^ g^-1^ Fw)	5.59±0.09c (5.31, 5.87)	7.80±0.30a (6.83, 8.76)	6.33±0.08b (6.09, 6.56)	6.77±0.21b (6.10, 7.44)
	GDH (nmol min^-1^ g^-1^ Fw)	0.14±0.10a (0.10, 0.18)	0.09±0.01b (0.07, 0.10)	0.09±0.01b (0.07, 0.12)	0.06±0.01c (0.03, 0.09)
	NH_2_-N content (mg g^-1^ Fw)	0.22±0.05a (0.07, 0.37)	0.14±0.03ab (0.03, 0.25)	0.10±0.01b (0.07, 0.13)	0.11±0.01ab (0.08, 0.15)
Stem	NH_4_^+^–N (μg g^-1^ Fw)	18.94±1.50d (14.16, 23.71)	44.99±3.50b (35.27, 54.71)	58.61±3.16a (48.56, 68.67)	34.28±2.82c (25.31, 43.25)
	NO_3_^-^–N (μg g^-1^ Fw)	345.31±46.37a (145.79, 544.82)	136.77±14.10b (76.13, 197.41)	234.63±19.53b (150.59, 318.66)	226.24±31.74b (125.24, 327.25)
	NR (nmol min^-1^ g^-1^ Fw)	167.18±16.18a (97.56, 236.80)	212.19±22.58a (115.03, 309.35)	102.88±19.64b (18.36, 187.40)	30.01±5.67c (5.61, 54.41)
	GS (μmol min^-1^ g^-1^ Fw)	4.86±0.29c (4.12, 5.61)	8.30±0.65b (6.64, 9.97)	13.10±0.38a (12.12, 14.07)	4.68±0.22c (4.06, 5.30)
	GDH (nmol min^-1^ g^-1^ Fw)	0.11±0.01ab (0.07, 0.14)	0.11±0.01a (0.08, 0.14)	0.08±0.00b (0.07, 0.10)	0.04±0.01c (0.03, 0.06)
	NH_2_-N content (mg g^-1^ Fw)	4.35±0.73a (1.21, 7.49)	3.62±0.37a (2.46, 4.78)	1.60±0.11b (1.12, 2.08)	2.21±0.28b (0.99, 3.44)

Nitrogen nutrient analysis in the roots and stems (**Table 3**) of *M. micrantha* and its companion plants revealed that *M. micrantha* exhibited the highest NO_3_^−^–N content in both roots and stems compared to other plants. Specifically, the NO_3_^−^–N content in *M. micrantha* roots was twice with non-overlapping 95% CI as high as in *I. nil* and *P. scandens*, while in stems, it was 1.4- and 1.5-fold higher than *I. nil* and *P. scandens*, respectively. The NH_4_^+^–N content was highest in the roots of *P. scandens*, while *I. nil* had the highest NH_4_^+^–N content in the stems.

Further analysis of enzyme activities involved in nitrogen metabolism (**Table 3**) revealed that *M. micrantha* had significantly higher NR and GDH activity in roots and stem compared to other plants. Specifically, NR activity in *M. micrantha* roots and stem was 2.1-/1.6- and 1.3-/5.6-fold higher than *I. nil* and *P. scandens*, respectively. GDH activity in *M. micrantha* roots was 1.6-, 1.5-, and 2.3-fold higher than *I. cairica*, *I. nil*, and *P. scandens.* In the stem, GDH activity in *M. micrantha* was similar to *I. cairica* but significantly higher than *P. scandens*. However, GS activity in *M. micrantha* was lower than that of other plants in both roots and stems.

As products of nitrogen metabolism, the free amino acid content in *M. micrantha* was also higher than in other plants, with the root content significantly higher than *I. nil*, approximately 2.2-fold. In stems, free amino acids were 2.7- and 2.0-fold higher than in *I. nil* and *P. scandens*, respectively.

### The change in phenotypic parameters of *M. micrantha* stems under different NO_3_^−^–N concentrations

**Figure 1:**
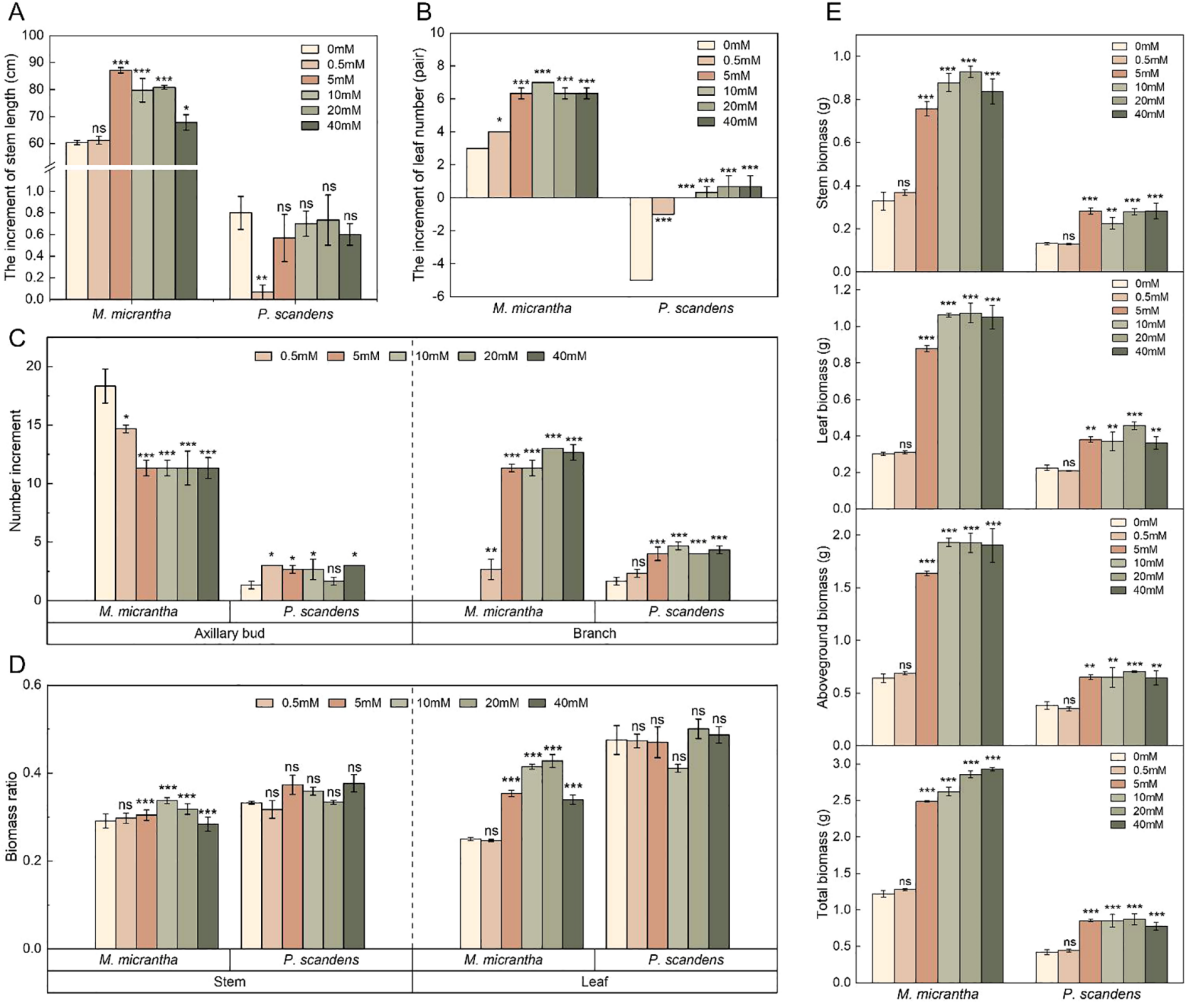
Change in growth indicators of *M. micrantha* and *P. scandens* under different NO_3_⁻-N concentrations at 30 days. **(A)** is the increment of stem length, **(B)** is the increment of leaf number, **(C)** is the increment of axillary bud and branch number, **(D)** is the stem and leaf biomass ratio, and **(E)** is the plant biomass, including the stem, leaves, aboveground biomass, and total biomass. The results are the mean ± SEM of five biological replicates. Each concentration group (0.5–40 mM) was compared with the control group (0 mM) by independent sample t-tests. Asterisks indicate significant differences (*p < 0.05, **p < 0.01, ***p < 0.001), and ns indicates no significant difference.

**Figure 2:**
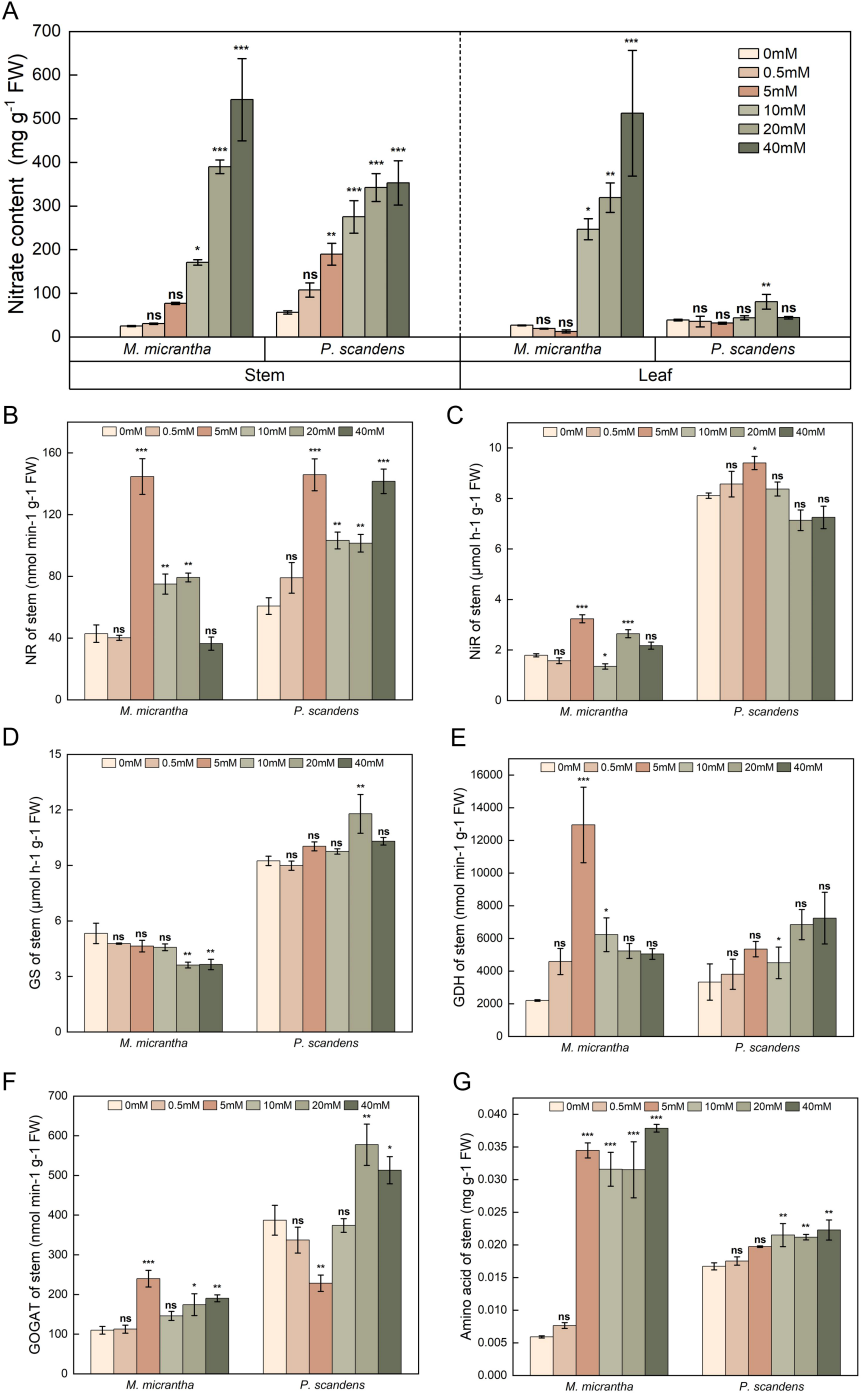
Changes in NO_3_^−^–N content and nitrogen metabolic enzyme Activity of *M. micrantha* and *P. scandens* under different NO_3_^−^–N concentrations at 30 days. **(A)** is the NO_3_^−^–N content in stem and leaf, **(B–G)** is the NR, NiR, GS, GDH, GOGAT activity and amino acid content in stem, respectively. The results are the mean ± SEM of five biological replicates. Each concentration group (0.5-40 mM) was compared with the control group (0 mM) by independent sample t-tests. Asterisks indicate significant differences (*p < 0.05, **p < 0.01, ***p < 0.001), and ns indicates no significant difference.

The results showed that there was a significant increase in the main stem length, leaf number and branch numbers of *M. micrantha* in the 5, 10, 20, and 40 mM treatment groups compared to the 0 mM (*p*<0.05). The increments in stem length for the 0.5, 5, 10, 20, and 40 mM groups were respectively 1-, 1.4-, 1.3-, 1.3-, and 1.1-fold (**Figure 1A**). Notably, the 5 mM group exhibits a significantly highest mean value compared to the 0 mM (**Supplementary Table S1**; mean difference (Δ) (95% CI of Δ) = 26.733 (19.720, 33.747), *p*<0.001); The increases in leaf numbers were 1.3-, 2.1-, 2.3-, 2.1-, and 2.2-fold, respectively (**Figure 2B**), in which the 10 mM group has the highest mean increment compared to the 0 mM (**Supplementary Table S1**; Δ(95% CI of Δ) = 4.000 (3.274, 4.726), *p*<0.001); and the increases in branch numbers were 2.7-, 11.3-, 11.3-, 13-, and 12.7-fold higher than the 0 mM group, with the 20 mM group exhibiting the highest mean value (**Supplementary Table S1**; Δ(95% CI of Δ) = 13.000 (11.323, 14.677), *p*<0.001), while axillary buds numbers significantly decreased (**Figure 1C**). In contrast, *P. scandens* showed no significant changes in stem length across treatments, as their 95% confidence intervals of mean difference include zero (**Supplementary Table S1**), though branching and axillary bud numbers increased compared to the control, with the exception of axillary buds in the 20 mM group. Overall, *M. micrantha* exhibited greater increases in stem length, leaf number, branch, and axillary bud numbers than *P. scandens* at the same NO_3_^−^–N concentrations, consistent with the phenotypic results (**Supplementary Figure S1**).

### The changes in biomass of different plant organs of *M. micrantha* stems under different NO_3_^−^–N concentrations

Further analysis of the biomass of the two plants showed that, compared to the 0 mM group, *M. micrantha* exhibited a significant increase in aboveground biomass and total biomass in the 5, 10, 20, and 40 mM treatment groups (**Figures 1C, E**), with the 10 mM group exhibiting the highest increment in aboveground biomass (**Supplementary Table S1**; Δ(95% CI of Δ) = 1.290 (1.046, 1.534), *p*<0.001). Specifically, the biomass values for stem, leaf, aboveground, and total biomass in *M. micrantha* were consistently higher across all treatments, with increases ranging from 1.1- to 2.8-fold for stem biomass, 1- to 3.5-fold for leaf biomass, 1.1- to 3-fold for aboveground biomass and 1.1- to 2.4-fold for total biomass. In contrast, *P. scandens* showed more moderate increases in biomass, with the highest increments in the 5 and 20 mM groups. Additionally, *M. micrantha* demonstrated an increase in leaf biomass ratio with the increase in NO_3_^−^–N concentration, while the stem biomass ratio remained relatively unchanged except for a 15.9% increase at 10 mM. Conversely, *P. scandens* showed no significant changes in stem and leaf biomass ratios under different treatments (**Figure 1D**).

### The changes in NO_3_^−^–N content and the expression levels of key genes involved in NO_3_^−^–N absorption and transport in *M. micrantha* stems under different NO_3_^−^–N concentrations

**Figure 3:**
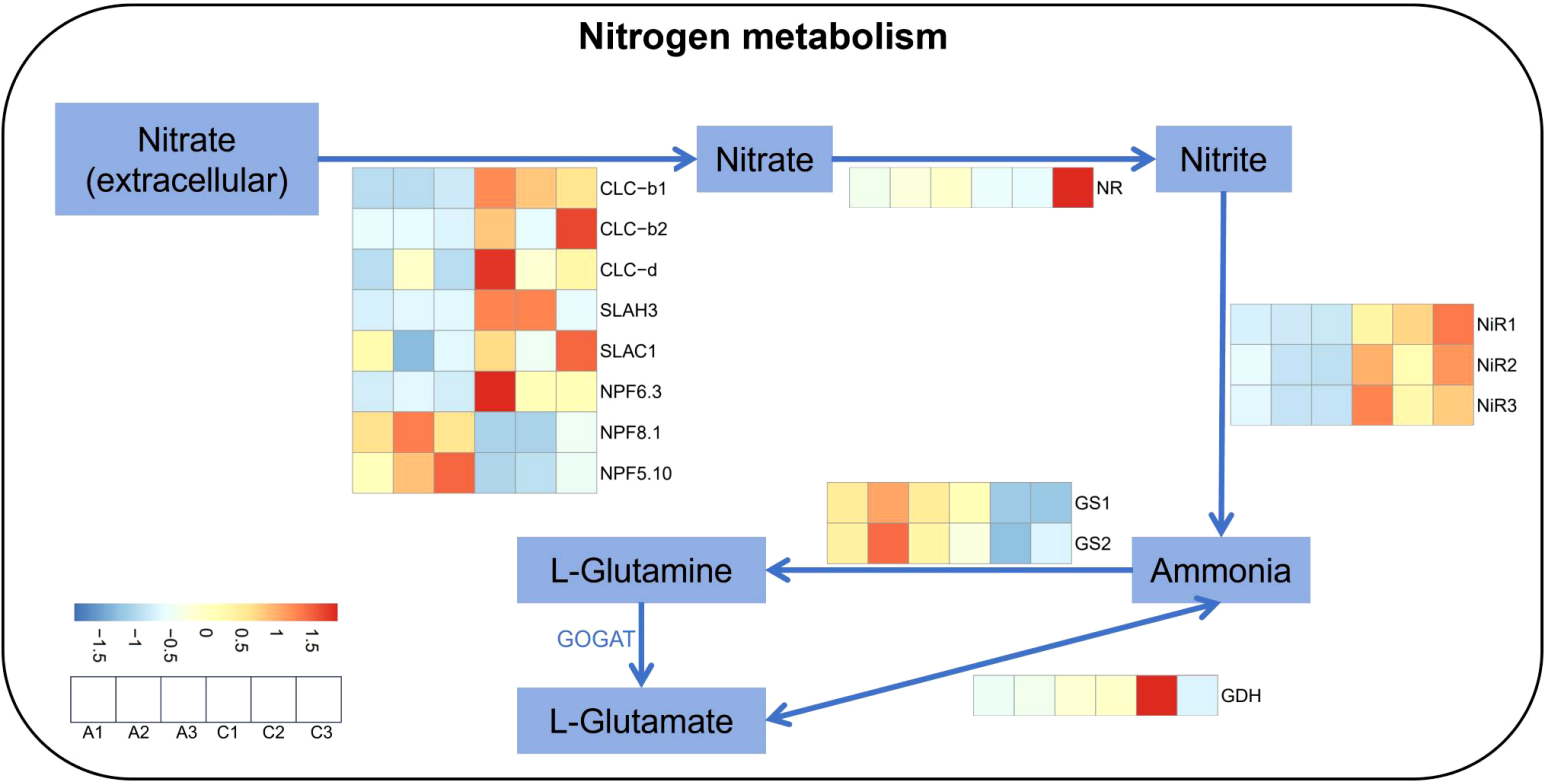
Heat map of genes related to nitrogen metabolism in *M. micrantha* stem under 0 mM and 5 mM NO_3_^−^–N treatment.

**Figure 4:**
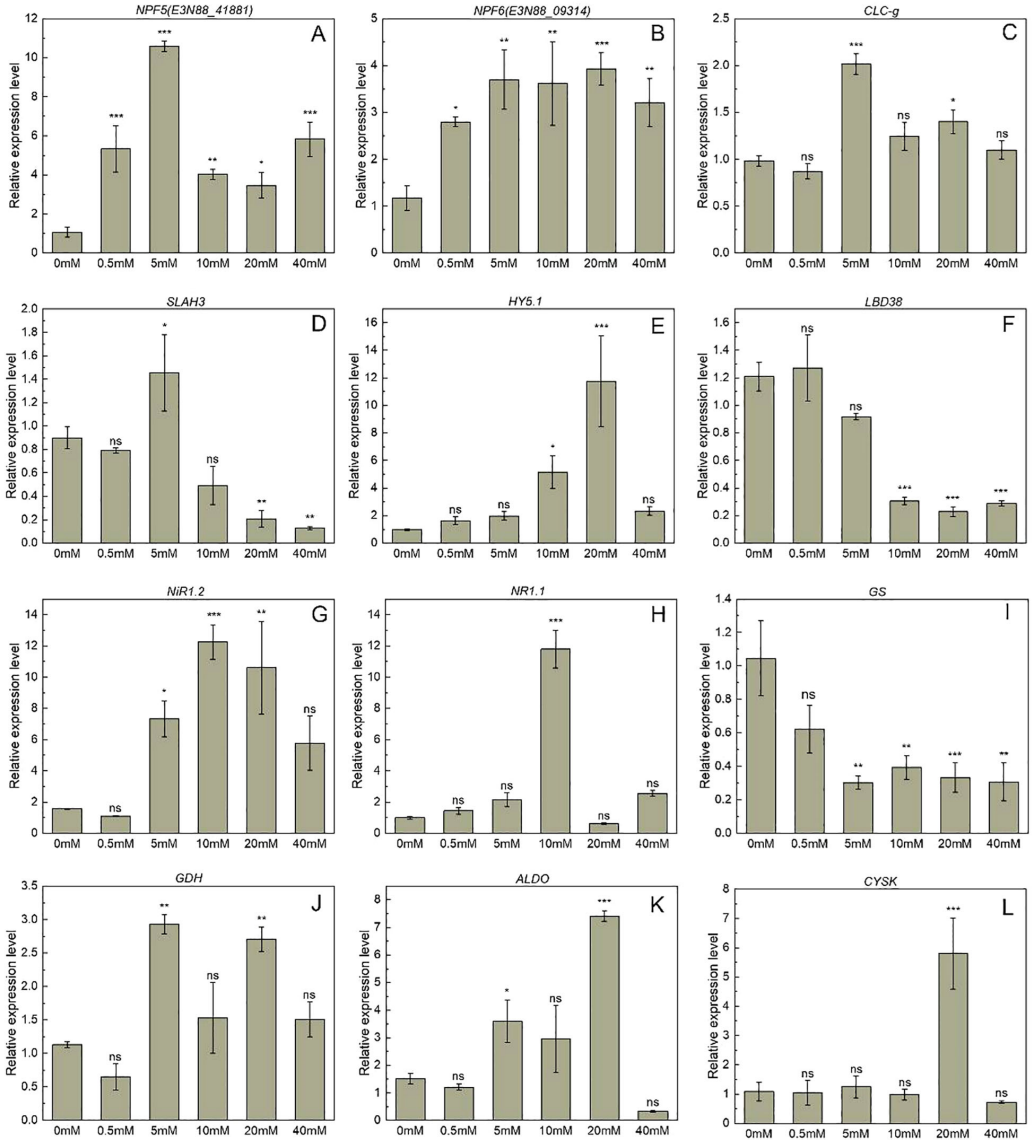
Gene expression related to nitrogen absorption transporters in *M. micrantha* stem under different NO_3_^−^–N concentrations. **(A, B)** are E3N88_41881 (*NPF5*), E3N88_09314 (*NPF6*), respectively. **(C–F)** are *CLC-g*, *SLAH3*, *HY5.1* and *LBD38*, respectively. **(G–J)** are *NiR1.2*, *NR1.1*, *Gs* and *GDH*, respectively. **(K, L)** are *ALDO* and *CYSK*, respectively. The results are the mean ± SEM of six biological replicates. Each concentration group (0.5-40 mM) was compared with the control group (0 mM) by independent sample t-tests. Asterisks indicate significant differences (*p < 0.05, **p < 0.01, ***p < 0.001), and ns indicates no significant difference.

The NO_3_^−^–N content in the stems of two plants was higher than that in the control group, with *M. micrantha* and *P. scandens* stems showing NO_3_^−^–N content 1.2-/1.9-fold, 3.1-/3.4-fold, 6.9-/4.9-fold, 15.7-/6.1-fold, and 21.9-/6.3-fold higher than the control group under 0.5, 5, 10, 20, and 40 mM treatments, respectively, suggesting that NO_3_^−^–N content of two species reached the highest level in the 40 mM group (**Supplementary Table S2**; Δ(95% CI of Δ) = 518.866 (398.579, 639.156), *p*<0.001/Δ(95% CI of Δ) = 296.782 (200.144, 393.421), *p*<0.001). Interestingly, the elevation in *M. micrantha* at 20 and 40 mM treatments were higher than that in *P. scandens*, as the higher mean values and mean differences of *M. micrantha* (**Figure 2A**; **Supplementary Table S2**). RNA-seq and qPCR results revealed that *M. micrantha* exhibited upregulation of genes related to NO_3_^−^–N absorption at 5 mM group. Notably, *NPF6.3*, *CLCb1*, *CLCb2*, and *SLAH3* showed the most significant upregulation, with expression levels 3.8- to 9.3-fold higher than 0 mM group, respectively. However, two genes in the NPF family, *NPF8.1* and *NPF5.10*, were significantly downregulated at 5 mM, with expression levels 0.48- and 0.17-fold of the control, respectively (**Figure 3**). QPCR results further demonstrated that under 0.5, 5, 10, 20, and 40 mM group, the expression levels of *NPF5* in *M. micrantha* were 3.3- to 10-fold of the 0 mM group (**Figure 4A**). The expression levels of *NPF6* in *M. micrantha* were 2.4- to 3.4-fold of the 0 mM group (**Figure 4B**). Further analysis showed varying expression patterns of transcription factors. Positive regulators, such as *CLC-g*, *HY5.1*, and *SLAH3*, exhibited the highest expression at different concentrations, with *CLC-g* (**Figure 4C**) and *SLAH3* (**Figure 4D**) peaking at 5 mM (2.1- and 1.6-fold, respectively), while *HY5.1* (**Figure 4E**) peaked at 20 mM (12-fold). The negative feedback factor *LBD38* decreased in expression with higher NO_3_^−^–N concentrations (**Figure 4F**), especially at 10, 20, and 40 mM, where expression levels were 0.3-, 0.2-, and 0.2-fold of the 0 mM group.

### The changes in nitrogen metabolism enzyme activities and the expression levels of key genes in nitrogen metabolism pathways in *M. micrantha* stems under different NO_3_^−^–N concentrations

In *M. micrantha*, NR activity significantly increased at 5, 10, and 20 mM NO_3_^−^–N concentrations compared to the 0 mM group, with 3.4-, 1.8-, and 1.9-fold increases, respectively, and a similar trend was observed in *P. scandens* (**Figure 2B**). NiR activity in *M. micrantha* was 1.8- and 1.5-fold higher in the 5 and 20 mM groups (**Supplementary Table S2**; Δ(95% CI of Δ) = 1.446 (1.051, 1.840), *p*<0.001/Δ(95% CI of Δ) = 0.853 (0.458, 1.247), *p*<0.001), while *P. scandens* showed a 16% increase at 5 mM (**Supplementary Table S2**; Δ(95% CI of Δ) = 1.296 (0.186, 2.406), *p*<0.05, **Figure 2C**). For the GS/GOGAT cycle, GS activity in *M. micrantha* significantly decreased in the 20 and 40 mM groups, while *P. scandens* showed no significant difference, except for a significant increase in the 20 mM group (**Figure 2D**). GOGAT activity in *M. micrantha* peaked at 5 mM, showing a 2.2-fold increase (**Supplementary Table S2**; Δ(95% CI of Δ) = 129.980 (79.608, 180.352), *p*<0.001), whereas *P. scandens* exhibited a significant decrease of 41.1% in the 5 mM group (**Supplementary Table S2**; Δ(95% CI of Δ) = -158.831 (-264.702, -52.960), *p*<0.01, **Figure 2F**). GDH activity in *M. micrantha* increased significantly at 5 mM (**Supplementary Table S2**; Δ(95% CI of Δ) = 10748.817 (7330.776, 14166.857), *p*<0.001), being 2.4-fold higher than in *P. scandens* (**Figure 2D**). Combining RNA-seq and qPCR results to further analyze genes related to nitrate assimilation, including NR, NiR, and GDH, showed high expression at 5 mM in *M. micrantha*. Notably, *NiR1/2/3* expression increased 21.6-, 10.7-, and 11-fold, respectively (**Figure 4G**). In contrast, *GS1* and *GS2* expression was low at 5 mM. *NR1.1* expression increased 11.8-fold at 10 mM (**Figure 4H**), while *NiR1.2* expression was significantly higher in the 5, 10, and 20 mM groups (4.6-, 7.6-, and 6.6-fold, respectively). GS expression decreased by 62.4–71.0% at higher NO_3_^−^–N concentrations (**Figure 4I**), while GDH gene expression increased by 159.2% at 5 mM (**Figure 4J**).

### The change in free amino acid content and the expression levels of key genes in amino acid synthesis pathways in *M. micrantha* stems under different NO_3_^−^–N concentrations

**Figure 5:**
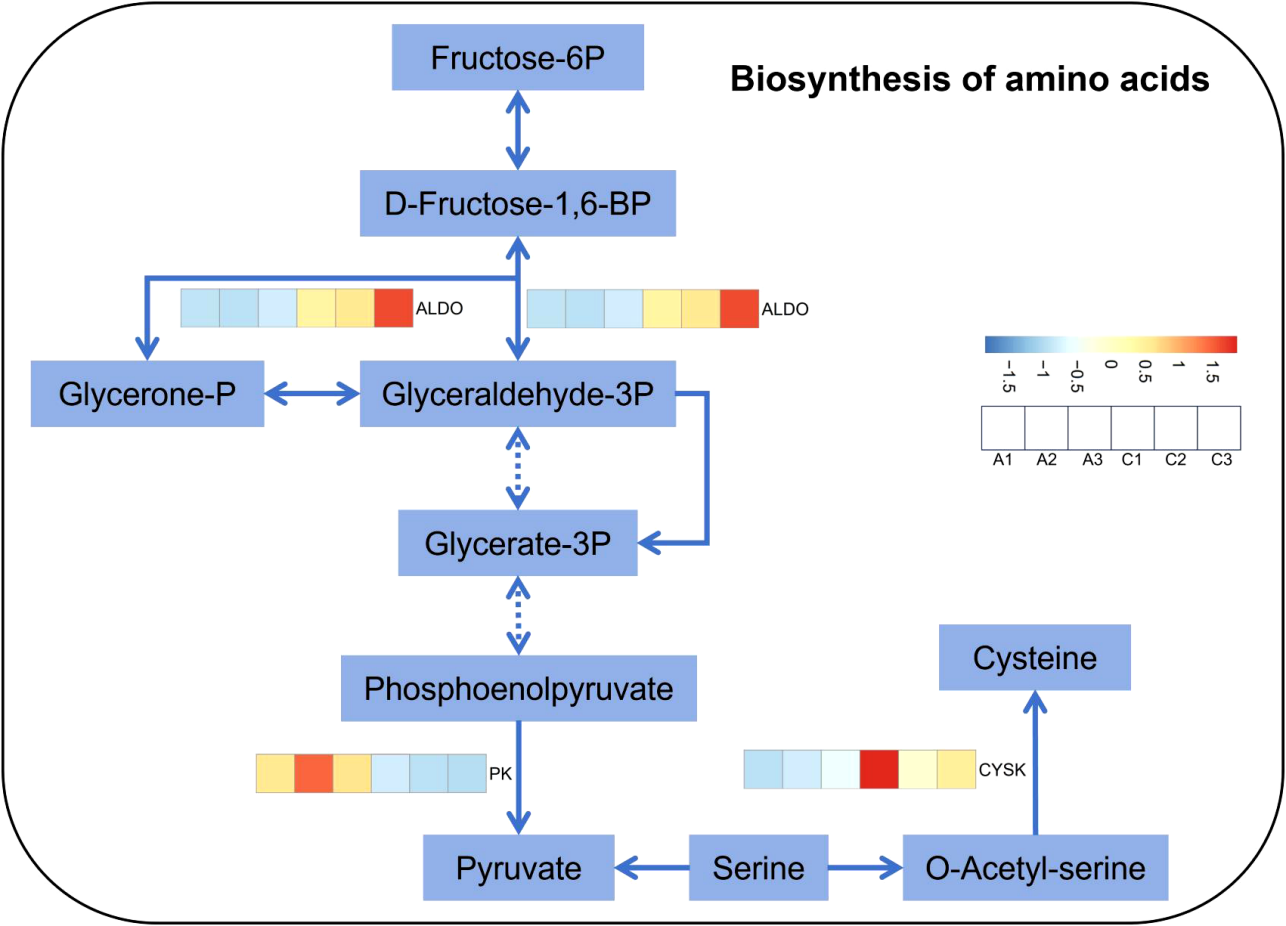
Heat map of genes related to biosynthesis of amino acids in *M. micrantha* stem under 0 mM and 5 mM NO_3_^−^–N treatment.

The free amino acid content in *M. micrantha* significantly higher under 5, 10, 20, and 40 mM treatments, with increases of 5.8-, 5.3-, 5.3-, and 6.3-fold, respectively. Similarly, *P. scandens* showed a significant increase in free amino acid content under 10, 20, and 40 mM treatments, with no significant difference under 0.5 and 5 mM treatments. Overall, *M. micrantha* exhibited higher free amino acid content than *P. scandens* under 5, 10, 20, and 40 mM treatments, as shown by the higher mean values and mean differences of *M. micrantha* (**Figure 2G**; **Supplementary Table S2**). Gene expression analysis revealed that the expression levels of *ALDO* and *CYSK* genes in *M. micrantha* was significantly higher at 5 mM group, approximately 2.5- and 3.8-fold of the 0 mM group, respectively. Conversely, *PK* gene expression was lower in the 5 mM group (**Figure 5**). qPCR results further demonstrated that under 0.5, 5, 10, 20, and 40 mM group, the expression levels of *ALDO* in *M. micrantha* were 0.8-, 2,4-, 2.0-, 4.9-and 0.2-fold of the 0 mM group, respectively (**Figure 4K**). The expression levels of *CYSK* in *M. micrantha* were 1.0-, 1.2-, 0.9-, 5.3- and 0.7-fold of the 0 mM group, respectively (**Figure 4L**).

### Correlation analysis between biomass and nitrogen metabolism-related enzyme activities

**Figure 6:**
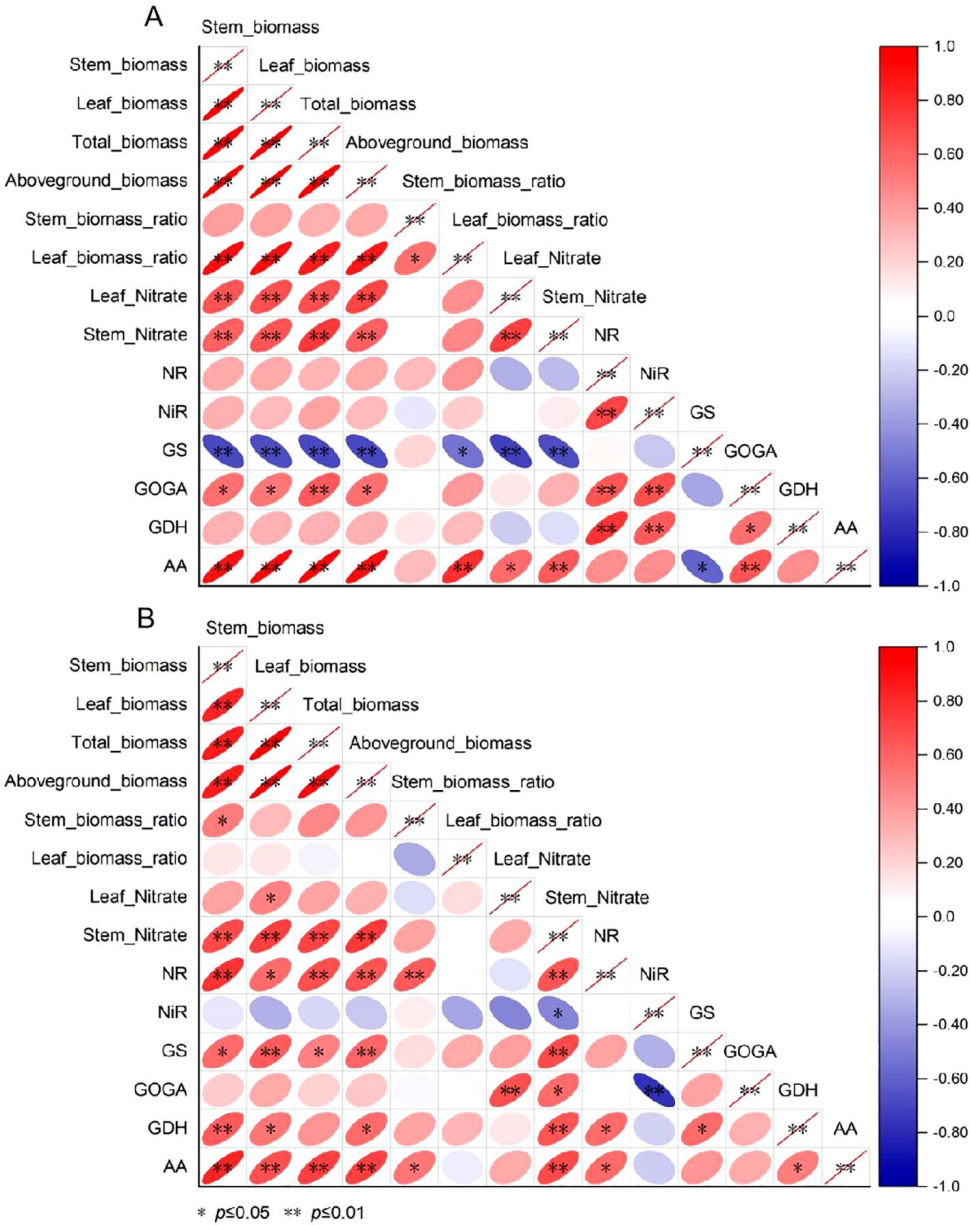
Correlation analysis between biomass and nitrogen metabolism-related enzyme activities in *M. micrantha* **(A)** and *P. scandens* **(B)**.

The correlation analysis revealed significant associations between biomass and nitrogen metabolism-related enzyme activities in two species. In *M. micrantha*, leaf biomass, stem biomass and total biomass displayed a highly positive correlation with NO_3_^−^–N content (**Figure 6A**). However, NO_3_^−^–N content had a relatively small impact on the stem biomass of *P. scandens*, and with no significant effect on leaf biomass (**Figure 6B**). Additionally, in *M. micrantha*, biomass demonstrated a significant positive correlation with GOGAT and FAA, a strong negative correlation with GS, and a positive correlation with NR and NiR but without statistical significance (**Figure 6A**). In *P. scandens*, biomass showed significant positive correlations with NR, Gs, GDH, and FAA, while presenting a negative correlation with NiR (**Figure 6B**).

### Principal component analysis

**Figure 7:**
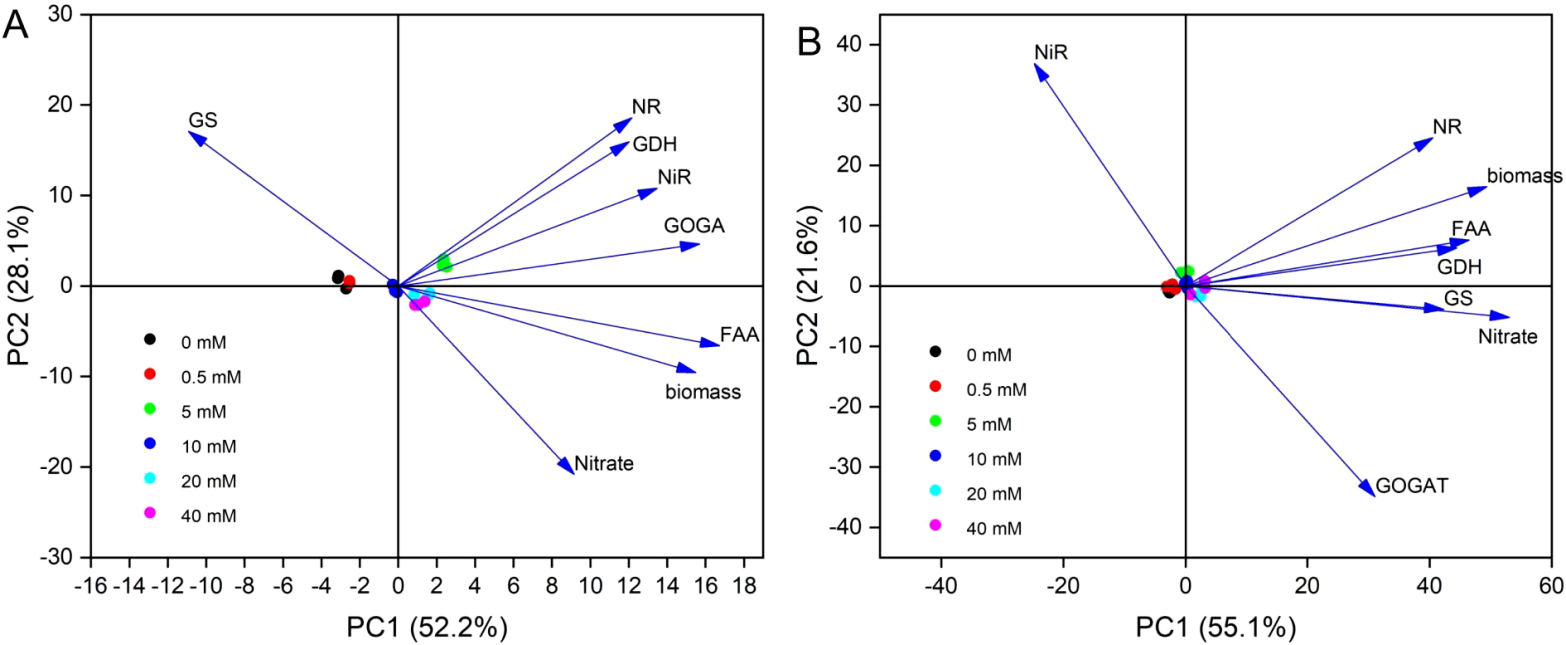
The principal component analysis (PCA) of in *M. micrantha* **(A)** and *P. scandens* **(B)**.

The scores plot showed that *M. micrantha* was significantly separated under different NO_3_^−^–N concentrations (**Supplementary Figure S5A**), suggesting its high sensitivity to changes in NO_3_^−^–N concentration and more variable metabolic response. The first two principal components (PC1 and PC2) accounted for 80.35% of the total variation. PC1 accounted for 52.21% of the total variance, with the highest contributions from Biomass (0.41), NiR (0.36), GOGAT (0.41) and FAA (0.44). These strongly correlated eigenvectors may affect stem growth by dynamically regulating nitrogen enzyme activities (NiR, GOGAT), thus explaining the rapid response to nitrate fluctuations. PC2 explained 28.14% of the total variance and was primarily influenced by Nitrate (-0.52), NR (0.46), GS (0.43), and GDH (0.40). The significant negative loading of NO_3_^−^–N and positive loadings of enzymes in PC2, potentially highlighting the influence of NO_3_^−^–N absorption and distribution on nitrogen metabolism (**Supplementary Table S3-1**). The PCA biplot reveals two distinct clustering groups. The 5 mM-40 mM groups were positively linked to NR, GDH, NiR, GOGAT, FAA, Biomass, and Nitrate, while the 0 mM-0.5 mM groups were positively associated with GS (**Figure 7A**). The diversity of biochemical traits in the 5 mM-40 mM group may suggest that *M. micrantha* responds to external nitrate fluctuations through flexible nitrogen assimilation strategy. Conversely, *P. scandens* clustered closely under different NO_3_^−^–N concentrations, indicating that it is insensitive to nitrate treatment and a relatively consistent response pattern (**Supplementary Figures S5B**, **Figure 7B**). PC1, which captures 55% of the variance, is largely influenced by Biomass (0.41), Nitrate (0.44), GS (0.35), GDH (0.37) and FAA (0.39), which may indicate that nitrogen metabolism (especially GS and GDH) possibly related to the insignificant changes in stem growth during increasing NO3^−^–N concentrations. PC2, which explains a smaller portion of the variance (21.59%), captures eigenvectors changes less directly related to responses under nitrate concentration (**Supplementary Table S3-2**).

## Discussion

### *M. micrantha* prefers to absorb NO_3_^−^–N

The observed variations in soil properties among *M. micrantha* and its companion plants not only highlight the influence of plant species on rhizospheric characteristics but also raise intriguing questions about the underlying mechanisms. The significant variations in NO_3_^−^–N and NH_4_^+^–N levels in the rhizospheric soil among different plant species underscore the complexity of nitrogen cycling in the root zone. The preferential uptake of NO_3_^−^–N by *M. micrantha*, as evidenced by the higher NO_3_^−^–N content in its rhizospheric soil (**Table 3**), aligns with the well-documented preference of certain plant species for specific nitrogen forms (Wang et al., 2018;Chen and Chen, 2019). Previous studies had shown that blackberry plants preferentially take up NH_4_^+^–N (Duan et al., 2023). *Sphagneticola canadensis* was a NO_3_^−^-N-preferring plant (Wang et al., 2023). The invasive plant *Xanthium strumarium* preferred to use NO_3_^−^, while its native congener *X. sibiricum* preferred to use NH_4_^+^ (Zhang et al., 2022). The preference of *Cymbidium tracyanum* for NO_3_^−^ as a nitrogen form may be a result of long-term adaptation to epiphytic habitat (Dong et al., 2024). The rhizospheric soil of *Bidens pilosa* (*Spanish needle*) exhibited a high concentration of NO_3_^−^–N, possibly attributed to the significant enhancement of soil nitrification by root exudates. This process converts more NH_4_^+^–N into absorbable NO_3_^−^–N, thereby increasing the root’s absorption efficiency of nitrogen nutrients and promoting the aboveground growth (Chen et al., 2009; Yu et al., 2021). Additionally, protease, as a crucial enzyme in the soil nitrogen cycle, influences the release of soil available nutrients. The rhizospheric soil of *M. micrantha* demonstrated elevated protease activity (**Table 3**), indicating an accelerated nitrogen metabolism process, thereby enhancing its efficiency in utilizing organic nitrogen (Li et al., 2006). Similar results have been observed in studies of other invasive plants such as *I. cairica*, *Synedrella nodiflora*, *Lantana camara*, and *W. trilobata*, where high protease activity in the rhizospheric soil was identified (Li et al., 2008).

### High expression of transporter genes promotes the absorption of NO_3_^−^–N

Nitrate is one of the main forms of inorganic nitrogen absorbed by plants, and its concentration variation has a direct impact on plant growth. Currently, research on the influence of NO_3_^−^–N on plant growth primarily focuses on aspects such as leaf photosynthetic capacity, leaf morphology, or root architecture (Takei et al., 2001; Crawford and Forde, 2002; Guiboileau et al., 2012; Vidal et al., 2014; Chen and Chen, 2019). However, limited attention has been given to non-foliar organs such as stems. Most studies have observed changes in physiological characteristics such as increased plant height, stem thickness, and biomass under higher nitrate nitrogen levels (Song et al., 2021), consistent with the findings of our study (**Figure 1**). The rapid growth of stems is one of the most prominent characteristics of *M. micrantha*. Our study found that with increasing NO_3_^−^–N concentration, the main stem length (**Figure 1A**) and leaf number (**Figure 1B**) of *M. micrantha* significantly increased, indicating that the axillary buds continuously activated the development of growth component branches (**Figure 1C**). As a result, the number of axillary buds decreased, while the number of branches increased (Bennett and Leyser, 2006). Results from PCA (**Figure 7**) and correlation analysis (**Figure 6**) further indicated that, unlike *P. scandens*, *M. micrantha* was more significantly influenced by NO_3_^−^–N, and its biomass was positively correlated with NO_3_^−^–N, NR, NiR, GOGAT, and FAA. The transport and assimilation of NO_3_^−^–N within plants directly impact plant growth and development, linked to their nitrogen metabolism capabilities (Yang et al., 2023; Xu et al., 2023). Currently, there was limited research on the nitrogen metabolism capacity of plant stems. RNA-seq analysis was conducted on *M. micrantha* stems treated with 5mM and 0 mM NO_3_^−^–N. Using Fold Change ≥ 2 and FDR < 0.01 as criteria for differential gene selection, 345 differentially expressed genes were identified, with 181 upregulated and 164 downregulated (**Supplementary Figure S2**). These genes primarily enriched pathways related to plant hormone signal transduction, phenylpropanoid biosynthesis, pentose and glucuronate interconversions, and nitrogen metabolism (**Supplementary Figure S4**). The results suggested associations between these pathways and nitrogen nutrient responses, indicating the involvement of some components in multiple nitrogen-related metabolisms, consistent with Wei et al. (2016) findings in Barley. The primary processes for plants to acquire and utilize NO_3_^−^–N include the absorption and transport of NO_3_^−^, reduction of NO_3_^−^ to NH_4_^+^, and assimilation of NH_4_^+^ into glutamic acid. Four gene families involved in NO_3_^−^–N absorption and transport have been discovered: *NRT1/NPF*, *NRT2*, *CLC*, and *SLAH*. These proteins are essential for the absorption, transport, and transfer of nitrate from the external environment to various cell types, tissues, and organs (Krapp et al., 2014). NO_3_^−^ induced the upregulation of *NPF6.3*, a nitrate transporter, which was primarily involved in transporting nitrate from the root to aboveground organs (Tsay et al., 2007). The expression of *NPF6.3* was significantly upregulated in the stem of *M. micrantha* (**Figure 3**), which played a role in transporting more NO_3_^−^–N for plant growth (**Figure 4A**) and as a signaling molecule regulating other physiological and ecological processes (Fan et al., 2017; Fredes et al., 2019). The high expression of *NPF5* genes (**Figure 4A**) in *M. micrantha* stems can regulate NO_3_^−^ balance between cytoplasm and vacuoles in response to changes in nitrogen supply (Wang et al., 2020), indicating that *NPF5* gene significantly enhances the nitrogen redistribution ability in plants and improves nitrogen utilization efficiency. In contrast, the expression of *NPF5.10* in *M. micrantha* stem was downregulated (**Figure 3**), which reduces the efflux of NO_3_^−^–N from the vacuole (Lu et al., 2022).

Additionally, the high expression of *CLC* and *SLAC* gene family members in *M. micrantha* (**Figure 3**) promotes NO_3_^−^–N transport within stems. Research suggested that these transporters also exhibited certain activity in NO_3_^−^ transport, altering the balance and storage of NO_3_^−^ and Cl^−^ in cells (Chopin et al., 2007; O’Brien et al., 2016). *M. micrantha* absorbed NO_3_^−^, and its transcription factor expression levels were correlated with its involvement in regulating NO_3_^−^, uptake. Under NO_3_^−^–N treatment, the transcription factor *HY5* in *M. micrantha*, involved in nitrate root signal regulation, showed increased expression levels at 10 mM and 20 mM NO_3_^−^–N treatments (**Figure 4E**). Previous research has highlighted the crucial role of *HY5* in regulating genes associated with nitrogen uptake and assimilation in plants (Mankotia et al., 2024). *HY5* positively regulates the expression of Nitrite reductase 1 (*NiR1*), known for converting nitrite into ammonium (Huang et al., 2015). Conversely, As a negative regulator, overexpression of *LBD* could have a certain inhibitory effect on nitrate responsive genes (*NRT1.1*, *NRT2.5*, etc.) (Rubin et al., 2009). Consistent with previous findings, the *LBD* gene family members in *M. micrantha* showed low expression levels under high NO_3_^−^–N treatment (**Figure 4F**). Therefore, the absorption and transport of NO_3_^−^–N are key steps in nitrogen absorption. The upregulation of key genes such as *NPF6.3*, *NPF5*, *CLC*, and *SLAC*, along with the regulatory roles of transcription factors like *HY5* and *LBD*, underscores the complexity of nitrogen absorption in *M. micrantha* compared to native plant, which was more conducive to its absorption of NO_3_^−^–N.

### Enhancing nitrogen metabolism capacity promotes the efficient assimilation of NO_3_^−^–N

The key enzymes involved in nitrogen metabolism play a crucial role in the nitrogen metabolism in plants. In *M. micrantha*, the expression levels of *NR*, *NiR*, and *GDH* were higher, while the expression of *GS* showed an opposite trend (**Figures 4G–J**, **3**), which was consistent with physiological data (**Figure 2**). These results indicated that a portion of NO_3_^−^ in the stem was being reduced, primarily through the GDH pathway for nitrogen assimilation, thus ensuring a higher nitrogen metabolism capacity. GDH and GS are key enzymes involved in ammonium assimilation in the nitrogen metabolism process. Previous studies have shown that nitrate can promote GS activity to some extent (Thu Hoai et al., 2003). However, under stress conditions, protein degradation produces a large amount of NH_4_^+^, which is prone to ammonia toxicity. The increase in GDH activity has a certain detoxification effect on NH_4_^+^ accumulation (Bittsánszky et al., 2015), which is similar to the opposite relationship with GS. With the increase of NO_3_^−^–N concentration, the NR (**Figure 2B**) and NiR (**Figure 2C**) activities in *M. micrantha* were higher, indicating that a large amount of NO_3_^−^ was gradually reduced to NH_4_^+^. To avoid the toxic effects of NH_4_^+^, the plant upregulated the expression of *GDH* gene (**Figure 4J**), enhancing GDH enzyme activity (**Figure 2E**), converting free NH_4_^+^ into glutamic acid and further converts it into other forms of organic nitrogen, providing precursors for the biosynthesis of nitrogen-containing compounds in plants. Therefore, GDH is considered the main nitrogen assimilation pathway in the stems of *M. micrantha.* Under high nitrogen conditions, with the increasing concentration of nitrate, the expression level of *GDH* was upregulated, indicating that *M. micrantha* optimizes nitrogen utilization by adjusting nitrogen metabolism pathways, thus enhancing its biomass and adaptability. This finding provides new insights into how *M. micrantha* responds to high-nitrogen environments. Similar studies (Yang et al., 2010) found that in cucumber seedlings, root ammonia assimilation was primarily accomplished through the GDH-induced pathway, while leaf ammonia assimilation was achieved through the GS/GOGAT cycle under nitrate treatment. However, the interaction between GDH and GS in ammonia assimilation in *M. micrantha* and how they balance the response to external NO_3_^−^ requires further research for in-depth exploration. GDH and GS play important roles in the assimilation process of *M. micrantha*, but further research is needed to balance the response to external NO_3_^−^ and increase more nutrients for plant growth. Positive regulatory genes (*ALDO* and *CYSK*) play a role in enhancing amino acid synthesis. It was observed that *M. micrantha* upregulated the expression levels of these two genes (**Figures 4KJ**, **7**), providing more precursors for amino acid synthesis, which was consistent with the significant increase in free amino acid content observed. Previous studies have suggested that tomato (*Solanum lycopersicum* L.) under low nitrogen conditions enhances the expression levels of *ALDO* gene to increase metabolism and obtain the substances or energy needed to adapt to environmental changes (Xu et al., 2023). Therefore, by regulating the expression levels of related transcription factors and nitrate transporter genes, *M. micrantha* enhances its ability to absorb nitrate nitrogen, improves nitrogen metabolism in the stem, and promotes the accumulation of a large number of amino acids, ensuring its higher biomass.

## Conclusion

*M. micrantha*, as a rapidly growing invasive plant, has garnered widespread attention and research. Our study analyzed physicochemical properties in rhizosphere soils, identified relevant differentially expressed genes, and constructed key transcriptional regulatory pathways. The results revealed that NO_3_^−^–N effectively promoted the growth of *M. micrantha*, including an increase in plant height, branching and biomass. Two potential mechanisms underlie these phenotypic changes in *M. micrantha* for better NO_3_^−^ acquisition. Firstly, compared to companion plants, *M. micrantha* exhibited higher NO_3_^−^–N content and protease activity in the soil. This process may accelerate nitrogen metabolism in the rhizosphere soil of *M. micrantha*, improving the efficiency of root utilization of nitrate nitrogen. Secondly, increased activities of NR, NiR, GS, and GOGAT in the stem enhanced nitrogen assimilation and amino acid biosynthesis, thereby promoting plant growth. In summary, under the backdrop of increasing global nitrogen deposition, particularly with the continued rise in NO_3_^−^–N deposition, the rapid growth of *M. micrantha* may be facilitated by the regulation of NO_3_^−^–N uptake transcription factors (*HY5*) and transport proteins (*CLC*, *SCLA/C*, *NPF*), as well as the expression regulation of key enzyme genes involved in nitrogen assimilation (*NR*, *GS*, *GOGAT*), thereby enhancing the nitrogen utilization efficiency of NO_3_^−^–N as the main form and accelerating the spread of *M. micrantha*. This study reveals the adaptation mechanisms of *M. micrantha* to NO_3_⁻-N enrichment, offering critical insights for predicting and managing invasive species responses to global atmospheric nitrogen deposition changes. The results highlight the importance of considering nitrogen composition, rather than just quantity, in invasive species management strategies.

## Data Availability

The original contributions presented in the study are included in the article/**Supplementary Material**. Further inquiries can be directed to the corresponding author.
