# Supplementary material for: Nitrate nitrogen uptake and metabolism in Mikania micrantha stem: insights into enhanced growth and invasiveness
Source: Front Plant Sci. 2025 May 2;16:1525303. doi: 10.3389/fpls.2025.1525303 (PMC12081409; doi:10.3389/fpls.2025.1525303)
Supplement: Supplementary file 1 [file DataSheet1.docx]

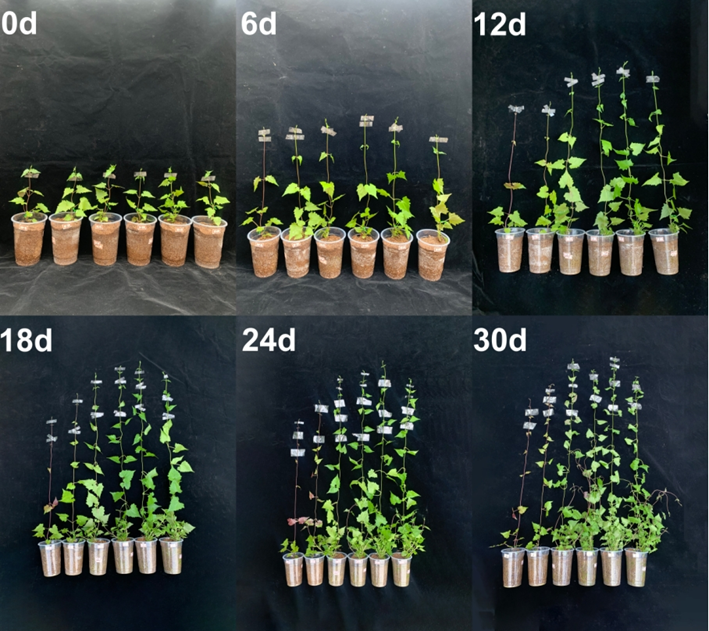


Figure S1-1 Phenotypic changes of *M. micrantha* under different NO_3_^−^–N concentrations with time. The plants on the picture are treated in the order of 0, 0.5 Mm, 5 mM, 10 mM, 20 mM and 40 mM NO_3_^−^–N.


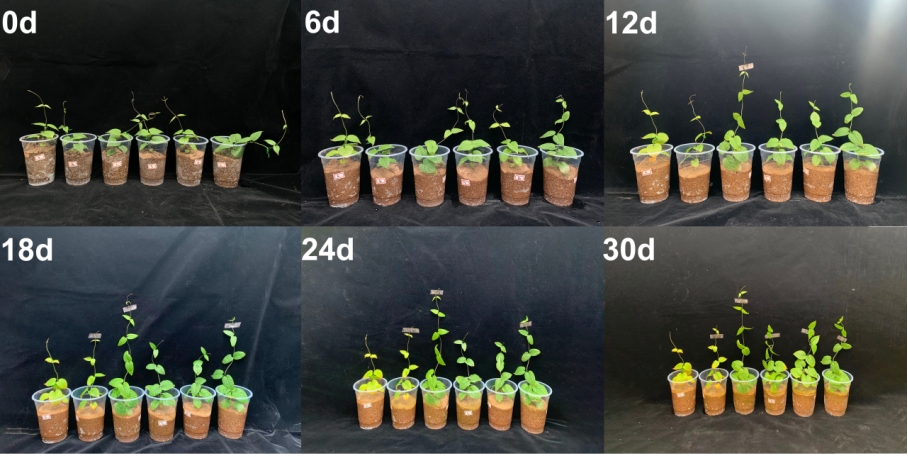


Figure S1-2 Phenotypic changes of *P. scandens* under different NO_3_^−^–N concentrations with time. The plants on the picture are treated in the order of 0, 0.5 Mm, 5 mM, 10 mM, 20 mM and 40 mM NO_3_^−^–N.
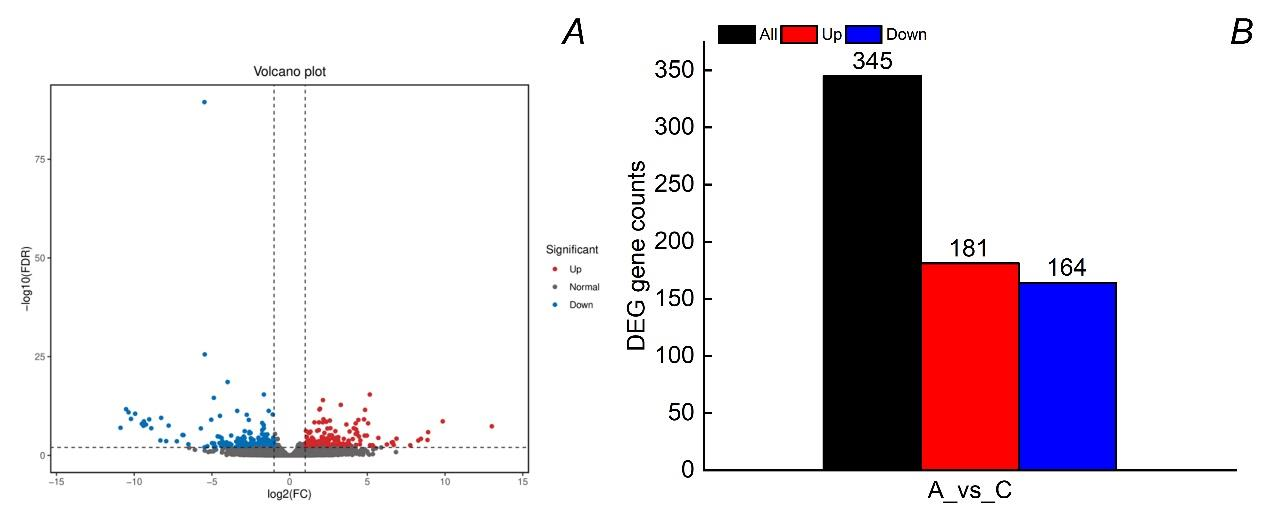


Figure S2 Statistics of differential genes. A represents the differential gene volcano map and B represents the number of differential genes.


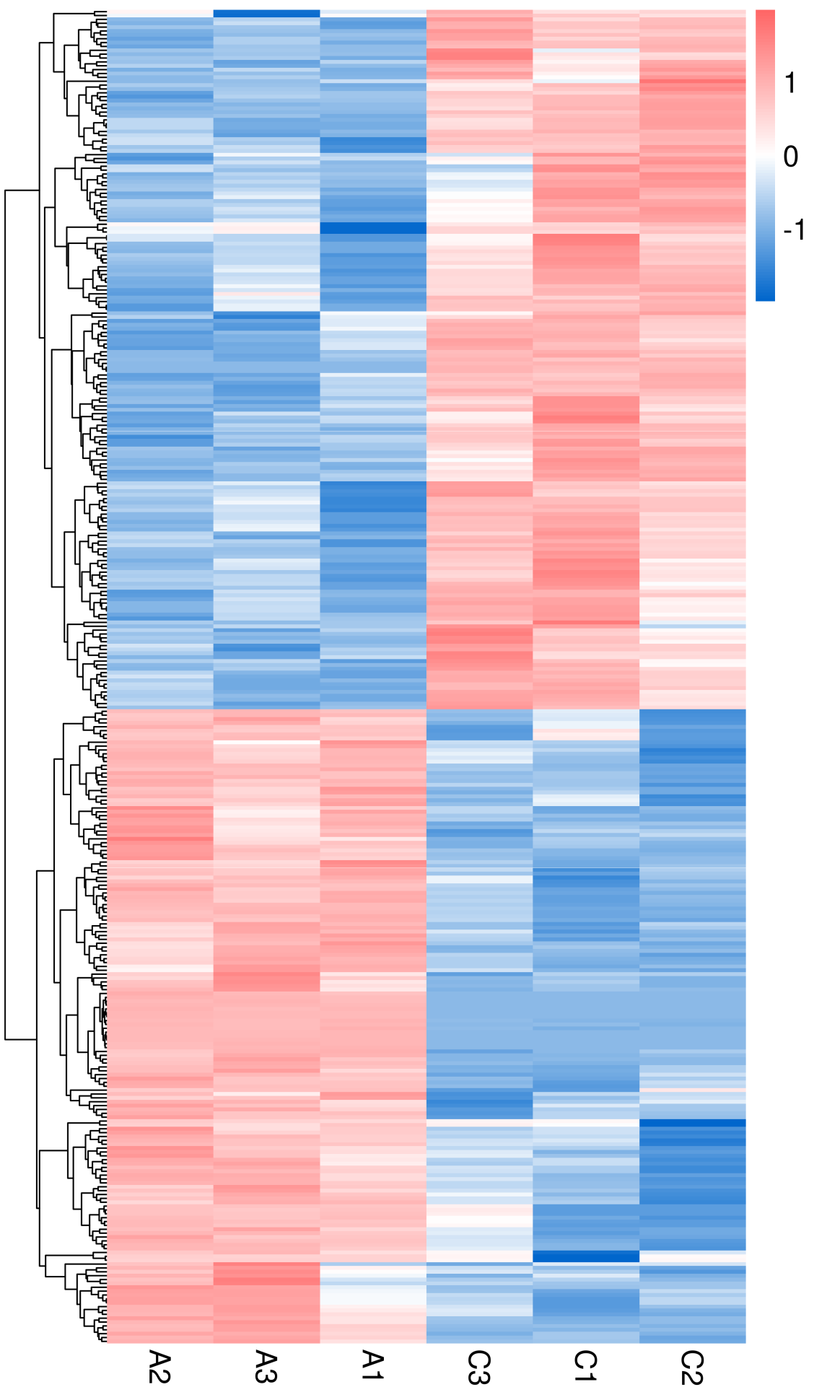


Figure S3 Heat map of genes related of 0 mM (A group) and 5 mM (C group) NO_3_^−^–N treatment.


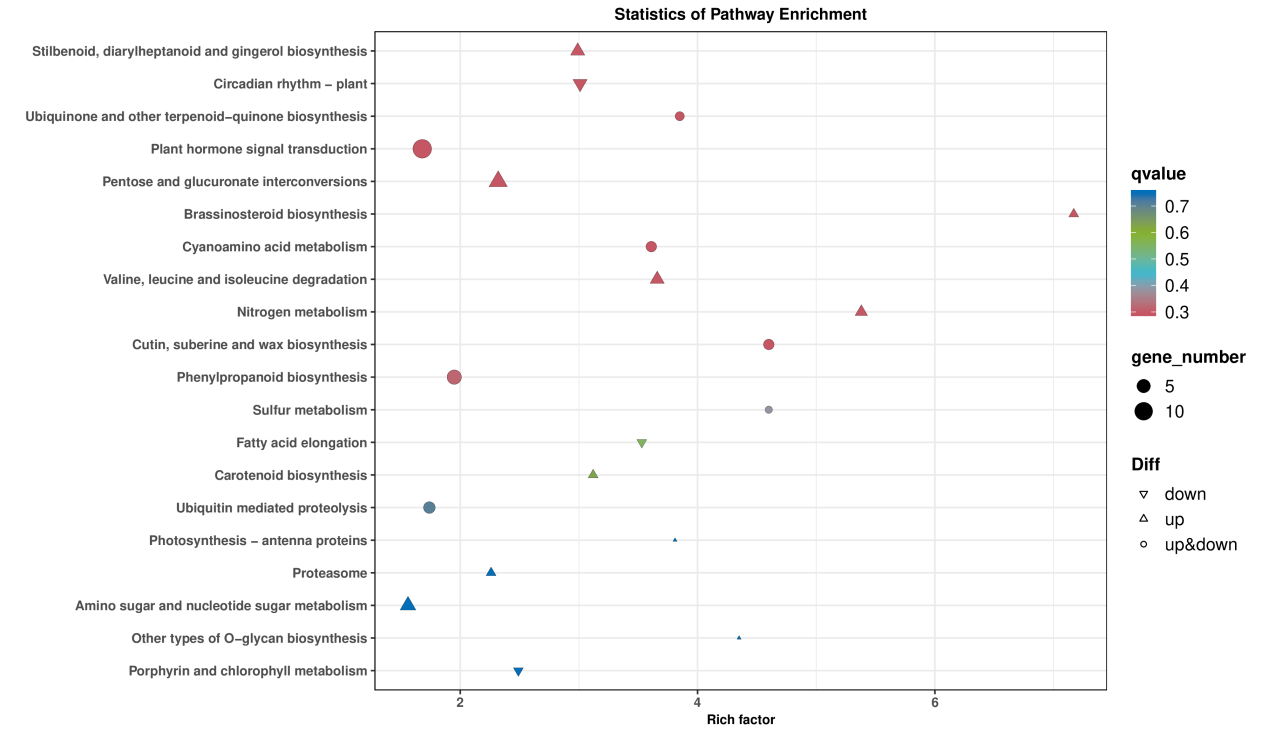


Figure S4 Enrichment results of differential gene KEGG pathway

Table S1 The statistical results of the relevant indicators in Figure 1.

| Indicators | Species | Concentrations  (mM) | Sample sizes | Mean | SEM | Mean difference  (Δ) | 95% CI of Δ | | *P* value |
| --- | --- | --- | --- | --- | --- | --- | --- | --- | --- |
|  |  |  |  |  |  |  | Lower | Upper |  |
| Stem length (Fig. 1A) | *M. micrantha* | 0 | 5 | 60.367 | 0.811 |  |  |  |  |
|  |  | 0.5 | 5 | 61.167 | 1.417 | 0.800 | -6.213 | 7.813 | 0.808 |
|  |  | 5 | 5 | 87.100 | 1.012 | 26.733* | 19.720 | 33.747 | 0.000 |
|  |  | 10 | 5 | 79.733 | 4.338 | 19.367* | 12.353 | 26.380 | 0.000 |
|  |  | 20 | 5 | 80.800 | 0.700 | 20.433* | 13.420 | 27.447 | 0.000 |
|  |  | 40 | 5 | 67.800 | 2.843 | 7.433* | 0.420 | 14.447 | 0.040 |
|  |  | | | | | | | | |
|  | *P. scandens* | 0 | 5 | 0.800 | 0.153 |  |  |  |  |
|  |  | 0.5 | 5 | 0.067 | 0.067 | -0.733* | -1.242 | -0.225 | 0.009 |
|  |  | 5 | 5 | 0.567 | 0.219 | -0.233 | -0.742 | 0.275 | 0.334 |
|  |  | 10 | 5 | 0.700 | 0.115 | -0.100 | -0.608 | 0.408 | 0.673 |
|  |  | 20 | 5 | 0.733 | 0.233 | -0.067 | -0.575 | 0.442 | 0.778 |
|  |  | 40 | 5 | 0.600 | 0.100 | -0.200 | -0.768 | 0.368 | 0.455 |
|  | | | | | | | | | |
| Leaf number (Fig. 1B) | *M. micrantha* | 0 | 5 | 3.000 | 0.000 |  |  |  |  |
|  |  | 0.5 | 5 | 4.000 | 0.000 | 1.000* | 0.274 | 1.726 | 0.011 |
|  |  | 5 | 5 | 6.333 | 0.333 | 3.333* | 2.607 | 4.060 | 0.000 |
|  |  | 10 | 5 | 7.000 | 0.000 | 4.000* | 3.274 | 4.726 | 0.000 |
|  |  | 20 | 5 | 6.333 | 0.333 | 3.333* | 2.607 | 4.060 | 0.000 |
|  |  | 40 | 5 | 6.333 | 0.333 | 3.333* | 2.607 | 4.060 | 0.000 |
|  |  | | | | | | | | |
|  | *P. scandens* | 0 | 5 | -5.000 | 0.000 |  |  |  |  |
|  |  | 0.5 | 5 | -1.000 | 0.000 | 4.000* | 2.742 | 5.258 | 0.000 |
|  |  | 5 | 5 | 0.000 | 0.000 | 5.000* | 3.742 | 6.258 | 0.000 |
|  |  | 10 | 5 | 0.333 | 0.333 | 5.333* | 4.075 | 6.591 | 0.000 |
|  |  | 20 | 5 | 0.667 | 0.667 | 5.667* | 4.409 | 6.925 | 0.000 |
|  |  | 40 | 5 | 0.667 | 0.667 | 5.667* | 4.409 | 6.925 | 0.000 |
|  | | | | | | | | | |
| Axillary bud number (Fig. 1C) | *M. micrantha* | 0 | 5 | 18.333 | 1.453 |  |  |  |  |
|  |  | 0.5 | 5 | 14.667 | 0.333 | -3.667* | -6.748 | -0.585 | 0.024 |
|  |  | 5 | 5 | 11.333 | 0.667 | -7.000* | -10.081 | -3.919 | 0.000 |
|  |  | 10 | 5 | 11.333 | 0.667 | -7.000* | -10.081 | -3.919 | 0.000 |
|  |  | 20 | 5 | 11.333 | 1.453 | -7.000* | -10.081 | -3.919 | 0.000 |
|  |  | 40 | 5 | 11.333 | 0.882 | -7.000* | -10.081 | -3.919 | 0.000 |
|  |  | | | | | | | | |
|  | *P. scandens* | 0 | 5 | 1.333 | 0.333 |  |  |  |  |
|  |  | 0.5 | 5 | 3.000 | 0.000 | 1.667* | 0.341 | 2.993 | 0.018 |
|  |  | 5 | 5 | 2.667 | 0.333 | 1.333* | 0.007 | 2.659 | 0.049 |
|  |  | 10 | 5 | 2.667 | 0.882 | 1.333* | 0.007 | 2.659 | 0.049 |
|  |  | 20 | 5 | 1.667 | 0.333 | 0.333 | -0.993 | 1.659 | 0.594 |
|  |  | 40 | 5 | 3.000 | 0.000 | 1.667* | 0.341 | 2.993 | 0.018 |
|  | | | | | | | | | |
| Branch number (Fig. 1C) | *M. micrantha* | 0 | 5 | 0.000 | 0.000 |  |  |  |  |
|  |  | 0.5 | 5 | 2.667 | 0.882 | 2.667* | 0.989 | 4.344 | 0.005 |
|  |  | 5 | 5 | 11.333 | 0.333 | 11.333* | 9.656 | 13.011 | 0.000 |
|  |  | 10 | 5 | 11.333 | 0.667 | 11.333* | 9.656 | 13.011 | 0.000 |
|  |  | 20 | 5 | 13.000 | 0.000 | 13.000* | 11.323 | 14.677 | 0.000 |
|  |  | 40 | 5 | 12.667 | 0.667 | 12.667* | 10.989 | 14.344 | 0.000 |
|  |  | | | | | | | | |
|  | *P. scandens* | 0 | 5 | 1.667 | 0.333 |  |  |  |  |
|  |  | 0.5 | 5 | 2.333 | 0.333 | 0.667 | -0.443 | 1.776 | 0.215 |
|  |  | 5 | 5 | 4.000 | 0.577 | 2.333* | 1.224 | 3.443 | 0.001 |
|  |  | 10 | 5 | 4.667 | 0.333 | 3.000* | 1.891 | 4.109 | 0.000 |
|  |  | 20 | 5 | 4.000 | 0.000 | 2.333* | 1.224 | 3.443 | 0.001 |
|  |  | 40 | 5 | 4.333 | 0.333 | 2.667* | 1.557 | 3.776 | 0.000 |
|  | | | | | | | | | |
| Stem biomass ratio (Fig. 1D) | *M. micrantha* | 0 | 5 | 0.291 | 0.016 |  |  |  |  |
|  |  | 0.5 | 5 | 0.298 | 0.012 | 0.007 | -0.033 | 0.046 | 0.726 |
|  |  | 5 | 5 | 0.305 | 0.012 | 0.013 | -0.026 | 0.053 | 0.479 |
|  |  | 10 | 5 | 0.338 | 0.007 | 0.046* | 0.007 | 0.086 | 0.026 |
|  |  | 20 | 5 | 0.318 | 0.012 | 0.027 | -0.013 | 0.067 | 0.165 |
|  |  | 40 | 5 | 0.284 | 0.016 | -0.007 | -0.047 | 0.032 | 0.696 |
|  |  | | | | | | | | |
|  | *P. scandens* | 0 | 5 | 0.333 | 0.003 |  |  |  |  |
|  |  | 0.5 | 5 | 0.317 | 0.020 | -0.015 | -0.062 | 0.032 | 0.490 |
|  |  | 5 | 5 | 0.373 | 0.022 | 0.041 | -0.006 | 0.088 | 0.083 |
|  |  | 10 | 5 | 0.359 | 0.009 | 0.026 | -0.021 | 0.073 | 0.247 |
|  |  | 20 | 5 | 0.334 | 0.005 | 0.001 | -0.046 | 0.048 | 0.963 |
|  |  | 40 | 5 | 0.377 | 0.020 | 0.044 | -0.003 | 0.091 | 0.064 |
|  | | | | | | | | | |
| Leaf biomass ratio (Fig. 1D) | *M. micrantha* | 0 | 5 | 0.250 | 0.004 |  |  |  |  |
|  |  | 0.5 | 5 | 0.247 | 0.002 | -0.004 | -0.030 | 0.022 | 0.767 |
|  |  | 5 | 5 | 0.354 | 0.007 | 0.103* | 0.077 | 0.129 | 0.000 |
|  |  | 10 | 5 | 0.415 | 0.006 | 0.164* | 0.138 | 0.190 | 0.000 |
|  |  | 20 | 5 | 0.428 | 0.014 | 0.177* | 0.151 | 0.203 | 0.000 |
|  |  | 40 | 5 | 0.340 | 0.011 | 0.089* | 0.063 | 0.115 | 0.000 |
|  |  |  |  |  |  |  |  |  |  |
|  | *P. scandens* | 0 | 5 | 0.475 | 0.033 |  |  |  |  |
|  |  | 0.5 | 5 | 0.473 | 0.016 | -0.002 | -0.076 | 0.072 | 0.951 |
|  |  | 5 | 5 | 0.470 | 0.035 | -0.005 | -0.079 | 0.069 | 0.882 |
|  |  | 10 | 5 | 0.411 | 0.009 | -0.064 | -0.138 | 0.010 | 0.085 |
|  |  | 20 | 5 | 0.501 | 0.022 | 0.025 | -0.049 | 0.100 | 0.470 |
|  |  | 40 | 5 | 0.487 | 0.019 | 0.012 | -0.063 | 0.086 | 0.740 |
|  | | | | | | | | | |
| Stem biomass (Fig. 1E) | *M. micrantha* | 0 | 5 | 0.329 | 0.041 |  |  |  |  |
|  |  | 0.5 | 5 | 0.368 | 0.013 | 0.040 | -0.078 | 0.157 | 0.477 |
|  |  | 5 | 5 | 0.757 | 0.033 | 0.428* | 0.311 | 0.545 | 0.000 |
|  |  | 10 | 5 | 0.878 | 0.042 | 0.549* | 0.431 | 0.666 | 0.000 |
|  |  | 20 | 5 | 0.928 | 0.027 | 0.560* | 0.482 | 0.717 | 0.000 |
|  |  | 40 | 5 | 0.836 | 0.057 | 0.507* | 0.390 | 0.625 | 0.000 |
|  |  | | | | | | | | |
|  | *P. scandens* | 0 | 5 | 0.132 | 0.006 |  |  |  |  |
|  |  | 0.5 | 5 | 0.129 | 0.004 | -0.003 | -0.065 | 0.060 | 0.922 |
|  |  | 5 | 5 | 0.283 | 0.014 | 0.151* | 0.088 | 0.214 | 0.000 |
|  |  | 10 | 5 | 0.226 | 0.027 | 0.094* | 0.031 | 0.156 | 0.007 |
|  |  | 20 | 5 | 0.279 | 0.015 | 0.147* | 0.084 | 0.209 | 0.000 |
|  |  | 40 | 5 | 0.282 | 0.036 | 0.150* | 0.087 | 0.213 | 0.000 |
|  | | | | | | | | | |
| Leaf biomass (Fig. 1E) | *M. micrantha* | 0 | 5 | 0.303 | 0.009 |  |  |  |  |
|  |  | 0.5 | 5 | 0.309 | 0.008 | 0.006 | -0.103 | 0.115 | 0.910 |
|  |  | 5 | 5 | 0.879 | 0.016 | 0.576* | 0.467 | 0.685 | 0.000 |
|  |  | 10 | 5 | 1.063 | 0.011 | 0.760* | 0.651 | 0.869 | 0.000 |
|  |  | 20 | 5 | 1.073 | 0.053 | 0.770* | 0.661 | 0.879 | 0.000 |
|  |  | 40 | 5 | 1.050 | 0.065 | 0.747* | 0.638 | 0.856 | 0.000 |
|  |  | | | | | | | | |
|  | *P. scandens* | 0 | 5 | 0.225 | 0.015 |  |  |  |  |
|  |  | 0.5 | 5 | 0.209 | 0.003 | -0.016 | -0.103 | 0.070 | 0.687 |
|  |  | 5 | 5 | 0.380 | 0.016 | 0.155* | 0.069 | 0.241 | 0.002 |
|  |  | 10 | 5 | 0.369 | 0.051 | 0.144* | 0.057 | 0.230 | 0.003 |
|  |  | 20 | 5 | 0.455 | 0.021 | 0.230* | 0.144 | 0.316 | 0.000 |
|  |  | 40 | 5 | 0.362 | 0.034 | 0.137* | 0.050 | 0.223 | 0.005 |
|  | | | | | | | | | |
| Aboveground biomass (Fig. 1E) | *M. micrantha* | 0 | 5 | 0.641 | 0.040 |  |  |  |  |
|  |  | 0.5 | 5 | 0.689 | 0.014 | 0.048 | -0.197 | 0.292 | 0.677 |
|  |  | 5 | 5 | 1.636 | 0.020 | 0.995* | 0.750 | 1.239 | 0.000 |
|  |  | 10 | 5 | 1.932 | 0.038 | 1.290* | 1.046 | 1.534 | 0.000 |
|  |  | 20 | 5 | 1.928 | 0.092 | 1.287* | 1.042 | 1.531 | 0.000 |
|  |  | 40 | 5 | 1.904 | 0.160 | 1.263* | 1.018 | 1.507 | 0.000 |
|  |  | | | | | | | | |
|  | *P. scandens* | 0 | 5 | 0.382 | 0.037 |  |  |  |  |
|  |  | 0.5 | 5 | 0.351 | 0.018 | -0.032 | -0.188 | 0.125 | 0.668 |
|  |  | 5 | 5 | 0.652 | 0.023 | 0.269* | 0.113 | 0.426 | 0.003 |
|  |  | 10 | 5 | 0.650 | 0.094 | 0.268* | 0.111 | 0.424 | 0.003 |
|  |  | 20 | 5 | 0.706 | 0.007 | 0.324* | 0.167 | 0.481 | 0.001 |
|  |  | 40 | 5 | 0.644 | 0.066 | 0.261* | 0.105 | 0.418 | 0.003 |
|  | | | | | | | | | |
| Total biomass (Fig. 1E) | *M. micrantha* | 0 | 5 | 1.220 | 0.047 |  |  |  |  |
|  |  | 0.5 | 5 | 1.280 | 0.015 | 0.059 | -0.061 | 0.180 | 0.305 |
|  |  | 5 | 5 | 2.485 | 0.012 | 1.264* | 1.144 | 1.385 | 0.000 |
|  |  | 10 | 5 | 2.623 | 0.061 | 1.402* | 1.281 | 1.523 | 0.000 |
|  |  | 20 | 5 | 2.857 | 0.048 | 1.637* | 1.516 | 1.758 | 0.000 |
|  |  | 40 | 5 | 2.930 | 0.024 | 1.709* | 1.588 | 1.830 | 0.000 |
|  |  | | | | | | | | |
|  | *P. scandens* | 0 | 5 | 0.420 | 0.037 |  |  |  |  |
|  |  | 0.5 | 5 | 0.444 | 0.021 | 0.024 | -0.146 | 0.194 | 0.763 |
|  |  | 5 | 5 | 0.854 | 0.016 | 0.434* | 0.264 | 0.603 | 0.000 |
|  |  | 10 | 5 | 0.853 | 0.087 | 0.433* | 0.263 | 0.602 | 0.000 |
|  |  | 20 | 5 | 0.872 | 0.075 | 0.452* | 0.283 | 0.622 | 0.000 |
|  |  | 40 | 5 | 0.777 | 0.053 | 0.357* | 0.187 | 0.527 | 0.001 |

Table S2 The statistical results of the relevant indicators in Figure 2.

| Indicators | Species | Concentrations  (mM) | Sample sizes | Mean | SEM | Mean difference  (Δ) | 95% CI of Δ | | *P* value |
| --- | --- | --- | --- | --- | --- | --- | --- | --- | --- |
|  |  |  |  |  |  |  | Lower | Upper |  |
| Nitrate content of stem (Fig. 2A) | *M. micrantha* | 0 | 5 | 24.844 | 1.047 |  |  |  |  |
|  |  | 0.5 | 5 | 30.229 | 1.562 | 5.385 | -114.904 | 125.673 | 0.924 |
|  |  | 5 | 5 | 76.596 | 2.498 | 51.752 | -68.536 | 172.040 | 0.367 |
|  |  | 10 | 5 | 170.677 | 6.316 | 145.833* | 25.545 | 266.122 | 0.022 |
|  |  | 20 | 5 | 390.100 | 15.751 | 365.257* | 244.968 | 485.545 | 0.000 |
|  |  | 40 | 5 | 543.712 | 94.054 | 518.866* | 398.579 | 639.156 | 0.000 |
|  |  | | | | | | | | |
|  | *P. scandens* | 0 | 5 | 56.270 | 3.527 |  |  |  |  |
|  |  | 0.5 | 5 | 107.423 | 16.305 | 51.154 | -45.484 | 147.792 | 0.271 |
|  |  | 5 | 5 | 189.688 | 25.150 | 133.419* | 36.781 | 230.057 | 0.011 |
|  |  | 10 | 5 | 275.244 | 37.271 | 218.974* | 122.336 | 315.613 | 0.000 |
|  |  | 20 | 5 | 342.252 | 31.868 | 285.983* | 189.345 | 382.621 | 0.000 |
|  |  | 40 | 5 | 353.052 | 50.855 | 296.782* | 200.144 | 393.421 | 0.000 |
|  | | | | | | | | | |
| Nitrate content of leaf (Fig. 2A) | *M. micrantha* | 0 | 5 | 26.639 | 1.047 |  |  |  |  |
|  |  | 0.5 | 5 | 19.011 | 1.047 | -7.628 | -195.935 | 180.679 | 0.931 |
|  |  | 5 | 5 | 12.579 | 3.517 | -14.060 | -202.367 | 174.247 | 0.873 |
|  |  | 10 | 5 | 246.660 | 24.019 | 220.021* | 31.714 | 408.329 | 0.026 |
|  |  | 20 | 5 | 319.053 | 33.689 | 292.414* | 104.107 | 480.722 | 0.005 |
|  |  | 40 | 5 | 512.600 | 143.813 | 485.962* | 297.654 | 674.269 | 0.000 |
|  |  | | | | | | | | |
|  | *P. scandens* | 0 | 5 | 38.605 | 1.686 |  |  |  |  |
|  |  | 0.5 | 5 | 35.314 | 12.268 | -3.291 | -30.640 | 24.058 | 0.798 |
|  |  | 5 | 5 | 31.575 | 2.023 | -7.030 | -34.379 | 20.319 | 0.586 |
|  |  | 10 | 5 | 43.840 | 4.701 | 5.235 | -22.114 | 32.584 | 0.684 |
|  |  | 20 | 5 | 80.784 | 16.858 | 42.179* | 14.831 | 69.528 | 0.006 |
|  |  | 40 | 5 | 43.989 | 2.991 | 5.385 | -21.964 | 32.734 | 0.676 |
|  | | | | | | | | | |
| NR of stem (Fig. 2B) | *M. micrantha* | 0 | 5 | 42.872 | 5.671 |  |  |  |  |
|  |  | 0.5 | 5 | 40.193 | 1.608 | -2.680 | -25.493 | 20.134 | 0.801 |
|  |  | 5 | 5 | 144.695 | 11.593 | 101.822* | 81.417 | 122.227 | 0.000 |
|  |  | 10 | 5 | 75.027 | 6.520 | 32.154* | 11.749 | 52.559 | 0.005 |
|  |  | 20 | 5 | 79.314 | 2.836 | 36.442* | 16.036 | 56.847 | 0.002 |
|  |  | 40 | 5 | 36.442 | 4.287 | -6.431 | -26.836 | 13.974 | 0.502 |
|  |  | | | | | | | | |
|  | *P. scandens* | 0 | 5 | 60.763 | 5.428 |  |  |  |  |
|  |  | 0.5 | 5 | 79.036 | 9.899 | 18.273 | -5.538 | 42.084 | 0.120 |
|  |  | 5 | 5 | 145.826 | 10.293 | 85.063* | 61.252 | 108.874 | 0.000 |
|  |  | 10 | 5 | 103.297 | 5.433 | 42.534* | 18.723 | 66.345 | 0.002 |
|  |  | 20 | 5 | 101.500 | 5.687 | 40.737* | 16.926 | 64.548 | 0.003 |
|  |  | 40 | 5 | 141.559 | 7.938 | 80.796* | 56.985 | 104.607 | 0.000 |
|  | | | | | | | | | |
| NiR of stem (Fig. 2C) | *M. micrantha* | 0 | 5 | 1.790 | 0.061 |  |  |  |  |
|  |  | 0.5 | 5 | 1.575 | 0.115 | -0.216 | -0.610 | 0.179 | 0.256 |
|  |  | 5 | 5 | 3.236 | 0.160 | 1.446* | 1.051 | 1.840 | 0.000 |
|  |  | 10 | 5 | 1.349 | 0.104 | -0.441* | -0.835 | -0.047 | 0.031 |
|  |  | 20 | 5 | 2.643 | 0.159 | 0.853* | 0.458 | 1.247 | 0.001 |
|  |  | 40 | 5 | 2.173 | 0.140 | 0.382 | -0.012 | 0.776 | 0.056 |
|  |  | | | | | | | | |
|  | *P. scandens* | 0 | 5 | 8.112 | 0.106 |  |  |  |  |
|  |  | 0.5 | 5 | 8.569 | 0.509 | 0.458 | -0.653 | 1.568 | 0.387 |
|  |  | 5 | 5 | 9.408 | 0.262 | 1.296* | 0.186 | 2.406 | 0.026 |
|  |  | 10 | 5 | 8.376 | 0.274 | 0.264 | -0.846 | 1.374 | 0.614 |
|  |  | 20 | 5 | 7.136 | 0.407 | -0.976 | -2.086 | 0.134 | 0.080 |
|  |  | 40 | 5 | 7.251 | 0.446 | -0.861 | -1.971 | 0.249 | 0.117 |
|  | | | | | | | | | |
| GS of stem (Fig. 2D) | *M. micrantha* | 0 | 5 | 5.326 | 0.551 |  |  |  |  |
|  |  | 0.5 | 5 | 4.778 | 0.034 | -0.548 | -1.471 | 0.376 | 0.221 |
|  |  | 5 | 5 | 4.641 | 0.314 | -0.685 | -1.608 | 0.239 | 0.132 |
|  |  | 10 | 5 | 4.573 | 0.181 | -0.753 | -1.676 | 0.170 | 0.101 |
|  |  | 20 | 5 | 3.614 | 0.157 | -1.711* | -2.635 | -0.788 | 0.002 |
|  |  | 40 | 5 | 3.649 | 0.280 | -1.677* | -2.601 | -0.754 | 0.002 |
|  |  | | | | | | | | |
|  | *P. scandens* | 0 | 5 | 9.246 | 0.256 |  |  |  |  |
|  |  | 0.5 | 5 | 8.991 | 0.250 | -0.255 | -1.714 | 1.204 | 0.710 |
|  |  | 5 | 5 | 10.031 | 0.241 | 0.786 | -0.673 | 2.245 | 0.263 |
|  |  | 10 | 5 | 9.757 | 0.144 | 0.511 | -0.947 | 1.970 | 0.460 |
|  |  | 20 | 5 | 11.790 | 1.047 | 2.544* | 1.085 | 4.003 | 0.003 |
|  |  | 40 | 5 | 10.307 | 0.204 | 1.061 | -0.398 | 2.520 | 0.139 |
|  | | | | | | | | | |
| GDH of stem (Fig. 2E) | *M. micrantha* | 0 | 5 | 2196.917 | 46.713 |  |  |  |  |
|  |  | 0.5 | 5 | 4586.733 | 799.308 | 2389.817 | -1028.224 | 5807.857 | 0.154 |
|  |  | 5 | 5 | 12945.733 | 2314.378 | 10748.817* | 7330.776 | 14166.857 | 0.000 |
|  |  | 10 | 5 | 6226.383 | 1035.199 | 4029.467* | 611.426 | 7447.507 | 0.025 |
|  |  | 20 | 5 | 5229.733 | 455.553 | 3032.817 | -385.224 | 6450.857 | 0.077 |
|  |  | 40 | 5 | 5047.550 | 326.287 | 2850.633 | -567.407 | 6268.674 | 0.094 |
|  |  |  |  |  |  |  |  |  |  |
|  | *P. scandens* | 0 | 5 | 3329.164 | 1107.458 |  |  |  |  |
|  |  | 0.5 | 5 | 3802.361 | 924.354 | 473.197 | -2757.723 | 3704.117 | 0.755 |
|  |  | 5 | 5 | 5344.007 | 470.917 | 2014.843 | -1216.077 | 5245.763 | 0.199 |
|  |  | 10 | 5 | 4502.886 | 966.218 | 1173.723 | -2057.197 | 4404.642 | 0.444 |
|  |  | 20 | 5 | 6843.070 | 923.957 | 3513.906* | 282.987 | 6744.826 | 0.035 |
|  |  | 40 | 5 | 7241.702 | 1583.312 | 3912.538* | 681.618 | 7143.458 | 0.022 |
|  | | | | | | | | | |
| GOGAT of stem (Fig. 2F) | *M. micrantha* | 0 | 5 | 109.880 | 9.663 |  |  |  |  |
|  |  | 0.5 | 5 | 112.560 | 10.117 | 2.680 | -47.692 | 53.052 | 0.910 |
|  |  | 5 | 5 | 239.860 | 21.060 | 129.980* | 79.608 | 180.352 | 0.000 |
|  |  | 10 | 5 | 146.060 | 11.449 | 36.180 | -14.192 | 86.552 | 0.144 |
|  |  | 20 | 5 | 174.200 | 27.495 | 64.320* | 13.948 | 114.692 | 0.017 |
|  |  | 40 | 5 | 190.280 | 8.787 | 80.400* | 30.028 | 130.772 | 0.005 |
|  |  | | | | | | | | |
|  | *P. scandens* | 0 | 5 | 387.180 | 37.491 |  |  |  |  |
|  |  | 0.5 | 5 | 337.087 | 32.560 | -50.094 | -155.965 | 55.777 | 0.323 |
|  |  | 5 | 5 | 228.349 | 20.693 | -158.831* | -264.702 | -52.960 | 0.007 |
|  |  | 10 | 5 | 373.949 | 17.196 | -13.231 | -119.102 | 92.640 | 0.790 |
|  |  | 20 | 5 | 577.453 | 52.074 | 190.273* | 84.402 | 296.144 | 0.002 |
|  |  | 40 | 5 | 513.208 | 34.379 | 126.028* | 20.157 | 231.899 | 0.023 |
|  | | | | | | | | | |
| Amino acid of stem (Fig. 2G) | *M. micrantha* | 0 | 5 | 0.006 | 0.000 |  |  |  |  |
|  |  | 0.5 | 5 | 0.008 | 0.000 | 0.002 | -0.005 | 0.008 | 0.573 |
|  |  | 5 | 5 | 0.034 | 0.001 | 0.029* | 0.022 | 0.035 | 0.000 |
|  |  | 10 | 5 | 0.032 | 0.003 | 0.026* | 0.019 | 0.032 | 0.000 |
|  |  | 20 | 5 | 0.032 | 0.004 | 0.026* | 0.019 | 0.032 | 0.000 |
|  |  | 40 | 5 | 0.038 | 0.001 | 0.032* | 0.025 | 0.039 | 0.000 |
|  |  | | | | | | | | |
|  | *P. scandens* | 0 | 5 | 0.017 | 0.001 |  |  |  |  |
|  |  | 0.5 | 5 | 0.018 | 0.001 | 0.001 | -0.002 | 0.004 | 0.588 |
|  |  | 5 | 5 | 0.020 | 0.000 | 0.003 | 0.000 | 0.006 | 0.061 |
|  |  | 10 | 5 | 0.021 | 0.002 | 0.005* | 0.002 | 0.008 | 0.007 |
|  |  | 20 | 5 | 0.021 | 0.000 | 0.004* | 0.001 | 0.008 | 0.010 |
|  |  | 40 | 5 | 0.022 | 0.002 | 0.006* | 0.002 | 0.009 | 0.003 |

Table S3-1. Eigenvalues of the principal component axes from the PCA of *M. micrantha.* Bold values indicate the characteristics that most influence each PC (|Eigenvalues| ≥ 0.30).

| **Eigenvectors** | **Components** | | |
| --- | --- | --- | --- |
|  | **1** | **2** | **3** |
| biomass | **0.41** | -0.24 | 0.46 |
| Nitrate | 0.24 | **-0.52** | -0.23 |
| NR | **0.32** | **0.46** | 0.16 |
| NiR | **0.36** | 0.27 | -0.64 |
| GS | -0.29 | **0.43** | 0.17 |
| GOGAT | **0.41** | 0.12 | -0.31 |
| GDH | **0.32** | **0.40** | 0.26 |
| FAA | **0.44** | -0.17 | 0.32 |
|  |  |  |  |
| *Eigenvalue* | *4.18* | *2.25* | *0.54* |
| *Variability (%)* | *52.21* | *28.14* | *6.74* |
| *Cumulative (%)* | *52.21* | *80.35* | *87.09* |

Table S3-2. Eigenvalues of the principal component axes from the PCA of *P. scandens.* Bold values indicate the characteristics that most influence each PC (|Eigenvalues| ≥ 0.30).

| **Eigenvectors** | **Components** | | |
| --- | --- | --- | --- |
|  | **1** | **2** | **3** |
| biomass | **0.41** | 0.27 | -0.21 |
| Nitrate | **0.44** | -0.09 | 0.02 |
| NR | **0.34** | **0.41** | -0.13 |
| NiR | -0.21 | **0.62** | 0.16 |
| GS | **0.35** | -0.07 | 0.66 |
| GOGAT | 0.26 | **-0.58** | -0.14 |
| GDH | **0.37** | 0.10 | 0.44 |
| FAA | **0.39** | 0.13 | -0.52 |
|  |  |  |  |
| *Eigenvalue* | *4.41* | *1.73* | *0.65* |
| *Variability (%)* | *55.14* | *21.59* | *8.08* |
| *Cumulative (%)* | *55.14* | *76.72* | *84.80* |


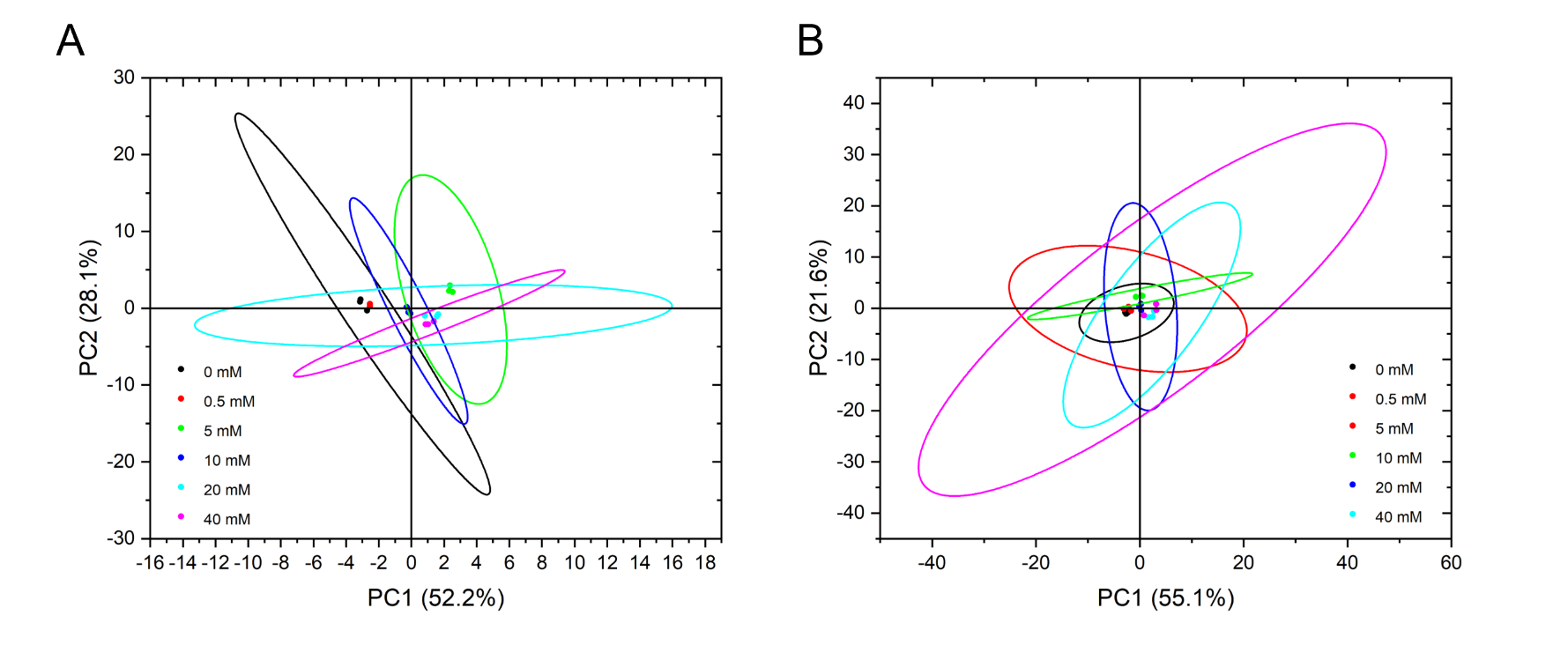


Figure S5. PCA score plot showing relationships among different NO_3_^−^–N concentrations of *M. micrantha* (A) and *P. scandens* (B).
